# The food-gut axis: lactic acid bacteria and their link to food, the gut microbiome and human health

**DOI:** 10.1093/femsre/fuaa015

**Published:** 2020-06-18

**Authors:** Francesca De Filippis, Edoardo Pasolli, Danilo Ercolini

**Affiliations:** Department of Agricultural Sciences, University of Naples Federico II, via Università, 100, 80055, Portici (NA) Italy; Task Force on Microbiome Studies, Corso Umberto I, 40, 80100, Napoli, Italy; Department of Agricultural Sciences, University of Naples Federico II, via Università, 100, 80055, Portici (NA) Italy; Task Force on Microbiome Studies, Corso Umberto I, 40, 80100, Napoli, Italy; Department of Agricultural Sciences, University of Naples Federico II, via Università, 100, 80055, Portici (NA) Italy; Task Force on Microbiome Studies, Corso Umberto I, 40, 80100, Napoli, Italy

**Keywords:** food microbiome, human microbiome, lactic acid bacteria, probiotics

## Abstract

Lactic acid bacteria (LAB) are present in foods, the environment and the animal gut, although fermented foods (FFs) are recognized as the primary niche of LAB activity. Several LAB strains have been studied for their health-promoting properties and are employed as probiotics. FFs are recognized for their potential beneficial effects, which we review in this article. They are also an important source of LAB, which are ingested daily upon FF consumption. In this review, we describe the diversity of LAB and their occurrence in food as well as the gut microbiome. We discuss the opportunities to study LAB diversity and functional properties by considering the availability of both genomic and metagenomic data in public repositories, as well as the different latest computational tools for data analysis. In addition, we discuss the role of LAB as potential probiotics by reporting the prevalence of key genomic features in public genomes and by surveying the outcomes of LAB use in clinical trials involving human subjects. Finally, we highlight the need for further studies aimed at improving our knowledge of the link between LAB-fermented foods and the human gut from the perspective of health promotion.

## Teaser

Lactic acid bacteria are widespread in food and many strains have a well-established role as probiotics; however, their occurrence, genome-wide diversity and role in the human gut and gut health is still understudied.

## INTRODUCTION

The lactic acid bacteria (LAB) group is phylogenetically located in the Clostridia branch of Gram-positive bacteria and includes non-sporing cocci, coccobacilli or rods, and aero-tolerant anaerobes, with a molar DNA base composition of less than 50% G + C (Pot *et al*. 1994). LAB are among the most widely studied microorganisms worldwide. Given the important role that LAB play in different biotechnological processes, it is not surprising that they have received much attention from the scientific community for decades. A search for the term ‘lactic acid bacteria’ in the title, keywords and abstract in the scientific database Scopus (www.scopus.com) (Burnham 2006) returned approximately 32,700 documents at the time of this review (May 2020). In addition, using ‘lactic acid bacteria’ AND ‘food’, ‘lactic acid bacteria’ AND ‘gut’ or ‘lactic acid bacteria’ AND ‘environment’ as search terms, 11,800, 1,500 and 1,700 documents can be retrieved, respectively, which clearly indicates that food is the most widely studied environment in association with LAB.

Although LAB exhibit considerable species and strain diversity and can play a significant role in different ecosystems, food remains their major source and preferred activity niche. This is mainly because the fermentation activity of LAB has been associated with foods and studied in fermented foods (FFs) since early 1900s. LAB activity in FFs can be basically considered a transformation of raw materials to edible food products with different characteristics. Food fermentation is actually an ancient process that was used as a strategy for food preservation, dating back to 10,000 years ago when agriculture and farming were introduced (Cordain *et al*. 2005). Food fermentation can be aerobic, such as alkaline fungal fermentation, and anaerobic, such as alcoholic and lactic acid fermentation by yeast and LAB, respectively (Nout 2014).

Some LAB strains are also considered potential probiotics, and many are commercialized in probiotic preparations and/or functional foods. In addition, they are also members of the gut microbiome of human and animal hosts, although their origin, role and potential activities are still widely discussed.

In this review, we discuss the occurrence of LAB species in both food and the human gut. Moreover, we assess the availability and information retrievable from available genomic and metagenomic data for LAB from food and humans. Finally, we discuss the effect of LAB on the gut microbiome on the basis of the currently available results from clinical trials and highlight future perspectives for exploiting the currently available genome-wide data that can help bridge the gap between food and the gut microbiome and can improve our understanding of the potential of FFs as vehicles for probiotic LAB.

### LAB diffusion and phylogenetic diversity

LAB are widely distributed in nutrient-rich habitats associated with food, plants, soil, animals and human hosts (Duar *et al*. 2017b; Wels *et al*. 2019). In recent years, the availability of a very large number of genomes of isolates from different sources has allowed comparative and evolutionary studies. Advancements made over the last few years were reviewed by (Duar *et al*. 2017b). In this work, the lifestyles of *Lactobacillus sensu lato* (i.e. including lactobacilli and related pediococci) were deduced by combining phylogenomic data with information about metabolism and data from the literature. The > 200 species (Sun *et al*. 2015) were first grouped in main clades based on the phylogeny according to Zheng *et al*. (2015a) and then assigned to three main clusters: free living (i.e. associated with plant material or the environment without relying on a eukaryotic host), host adapted (i.e. specialized for living in association with eukaryotic hosts, with adaptive traits that facilitate persistence), and nomadic (i.e. with a dynamic, generalist lifestyle that involves both environmental and host niches, with no signs of specialization) (Martino *et al*. 2016). Interestingly, lifestyle allocation overlaps with phylogenetic grouping at both the species and subspecies levels, suggesting the occurrence of adaptive genomic evolution in different niches (Duar *et al*. 2017). To elaborate, the *Lb. brevis, Lb. buchneri, Lb. collinoides, Lb. perolens, Lb. sakei*, and *Lb. vaccinostercus* groups were composed of species rarely found in animals and human hosts and therefore considered free living. Among the groups found to be nomadic, those species that, although not strictly autochthonous, exhibited adaptation to niches associated with humans or animals that could contribute to their persistence are of interest. These species could adapt to the gut and persist for at least a limited duration (Duar *et al*. 2017b). This is the case for *Lb. casei/paracasei* (Cai et al. 2007, 2009; Broadbent *et al*. 2012), *Lb. plantarum* (Siezen *et al*. 2010; Martino *et al*. 2016), and *Lb. rhamnosus* (Ribbera *et al*. 2013; Ceapa et al. 2015, 2016). *Lb. amylovorus, Lb. iners, Lb. johnsonii, Lb. reuteri, Lb. ruminis*, and *Lb. salivarius* were found to be adapted to vertebrate hosts, although some of them are also relevant in food fermentation (Vogel *et al*. 1999; Zheng *et al*. 2015b). The *Lb. delbrueckii* group comprised two main subclusters, one adapted to insects (e.g. *Lb. bombicola, Lb. apis*) and the other one to vertebrates (e.g. *Lb. johnsonii, Lb. gasseri*). Finally, the vertebrate gut was proposed as the real habitat of *Lb. helveticus*, despite its wide use in cheese production. Notably, host-adapted species or strains may have high ecological fitness in their respective hosts and therefore may be highly competitive when administered as probiotics (Duar *et al*. 2017a,b). On the other hand, species that did not undergone joint evolution with the host may be more appropriate for stimulating the immune system (Duar *et al*. 2017b). Notably, the lifestyle of pediococci remains unknown (Duar *et al*. 2017b), a problem that also exists for other LAB species that diverged from lactobacilli. The genus *Streptococcus* includes several pathogenic species, but the main food-related species, *Streptococcus thermophilus*, must have followed a divergent evolutionary path from that of its pathogenic relatives, and its genome has adapted to a well-defined and constant ecological niche, milk (Bolotin *et al*. 2004). This led to the loss of virulence factors and genes involved in the utilization of different carbohydrates, with the organism adapting to an environment in which the main carbohydrate source is lactose (Bolotin *et al*. 2004). Within the *Lactococcus* genus, *Lc. lactis* is of primary importance in the food industry. *Lc. lactis* taxonomy is currently based on phenotypic differentiation of two subspecies, *lactis* and *cremoris*. While the *cremoris* phenotype was found exclusively in dairy products and related environments, strains within the *lactis* subspecies have been isolated from different sources, including plants, vegetables and dairy environments (Wels *et al*. 2019). However, in *Lc. lactis* subsp. *cremoris*, a discrepancy between phenotype and genomic clustering was observed, and studies have shown that some strains with a *cremoris* genotype show a phenotype more similar to that of the *lactis* subspecies (Wels *et al*. 2019).

Other important LAB members are part of the family *Leuconostocaceae*, that includes heterofermentative microbes belonging to the genera *Leuconostoc, Weissella, Oenococcu*s and *Fructobacillus*. *Oenococcus* and *Fructobacillus* were originally assigned to *Leuconostoc* genus, but were reclassified later, while *Weissella* includes several species previously classified as *Lactobacillus* or *Leuconostoc* spp. The genus *Leuconostoc* have been isolated from different environments, including plant material, roots, clinical sources and fermented foods, mainly vegetables and dairy, as well as chilled raw meat, where they may act as spoilage agents (Holland and Liu 2011).

Taxonomic classification has been traditionally based on phenotypic traits and sugar metabolism profiling, and subsequently coupled with 16S rRNA gene sequencing. However, the introduction of novel species, together with the widespread of genomic technologies, highlighted that the current LAB taxonomy should be revised. Indeed, several works based on genomic comparison showed that genetic similarity within *Lactobacillus* genus is as low as the value usually found for different orders or even classes (Sun *et al*. 2015; Parks *et al*. 2018; Salvetti *et al*. 2018). In addition, members of other genera (e.g. *Pediococcus, Leuconostoc, Weissella, Oenococcus*) were shown to be intermixed among *Lactobacillus* species (Sun *et al*. 2015; Salvetti *et al*. 2018). Therefore, the *Lactobacillus* genus was proposed to be separated into 10 to 16 different genera (Pot *et al*. 2019). More recently, Zheng *et al*. (2020) showed that *Lactobacillaceae* and *Leuconostocaceae* families should be merged and suggested a reclassification of the genera included in these two families. Specifically, the emended *Lactobacillus* genus should incorporate only those species included in the *Lb. delbrueckii* group, while they proposed 25 novel genera enclosing other *Lactobacillus* species.

Although the urgent need for a reclassification was frequently highlighted and endorsed by an expert committee organised by the Lactic Acid Bacteria Industrial Platform (LABIP), a final decision was not taken yet. The renaming might have a strong impact on industry, consumers, regulators, as well as on the scientific and medical communities (Pot *et al*. 2019).

## FERMENTED FOODS, PROBIOTIC LAB AND FUNCTIONAL FOODS

By transforming carbohydrates provided by the raw materials to mainly lactic acid, LAB have contributed to food quality and safety for decades, although this has occurred with highly variable degrees of human awareness. In fact, knowledge of the actual contribution and potential of LAB in food fermentation has evolved over time, from popular but uninformed use of fermentation to well-thought-out selection and application of LAB as starter cultures for the food industry.

FFs can be split into at least two major categories: (i) industrial and (ii) artisanal. In the first case, appropriately selected LAB cultures are employed as starter cultures to assure the technological outcome of the fermentation and in some other cases are used as specialized ‘adjuncts’ that are able to perform specific metabolic activities that support aroma production or texture development or add further value to the food product (Burns *et al*. 2012). The selected LAB cultures are meant to help achieve high reproducibility, quality and safety in highly controlled fermentation. Conversely, artisanal food fermentation is usually carried out with no starter or with naturally selected cultures. In the absence of starter addition, the LAB of environmental origin available in the raw materials can take guide fermentation and assist in product manufacturing and in obtaining the final FF. Although the composition of natural starter cultures is considerably influenced by the specific product and type of fermentation, these cultures are composed mainly of various species and strains of LAB that are specifically and naturally selected by the manufacturing process and whose composition is heavily influenced by raw materials and technological as well as environmental conditions. The spontaneously selected LAB in natural starter cultures are selected by a series of inoculation and refreshment steps in a traditional back-slopping procedure, where part of the fermented matrix of a previous manufacturing process is used as a natural starter in the fermentation process on the following day. The high diversity of FFs available across the globe is mirrored by the equally high microbial diversity of LAB employed daily in food fermentation.

The wide variety of raw material-microbe combinations results in thousands of different FFs and fermented beverages (Marco *et al*. 2017). Milk, meat, fish, vegetables, cereals and legumes can be fermented to obtain a variety of end products of high quality. Although the industrial use of selected LAB cultures has improved speed and quality standards, the number of FFs available and their associated microbial diversity has reduced. However, many countries across the world are currently promoting the use of FFs, especially traditional foods, for both their hedonic (Xiang *et al*. 2019) and health-promoting properties (Chilton, Burton and Reid 2015).

Functional foods deliver additional or enhanced benefits over and above their basic nutritional benefits (Bell *et al*. 2018). LAB can contribute to rendering a FF functional, via both their presence and specific activities. While transforming raw materials through fermentation, LAB activity can indirectly confer several properties to FFs, making them valuable products for human health. In fact, beyond lactic and other acids, some metabolic activities and products can be developed during fermentation and confer interesting potential health-promoting properties to FFs (Şanlier, Gökcen and Sezgin 2019). Several observational studies have been performed to support this hypothesis and have linked the consumption of FFs (mostly yogurt) with beneficial effects on weight management (Mozaffarian *et al*. 2011), cardiovascular disease and type 2 diabetes (Chen *et al*. 2014; Tapsell 2015). Moreover, a link between FF consumption and mood and brain activity is also emerging (Tillisch *et al*. 2013; Aslam *et al*. 2018).

Several functional foods are recognized as such because they contain and deliver probiotic microorganisms. Many species and strains of LAB are regarded as probiotics, which are ‘live microorganisms that, when administered in adequate amounts, confer a health benefit on the host’, according to the definition proposed in 2001 by an expert panel working on behalf of the Food and Agriculture Organization of the United Nations and the World Health Organization (FAO/WHO) and subsequently endorsed by the International Scientific Association for Probiotics and Prebiotics (ISAPP) in the consensus statement of 2014 (Hill *et al*. 2014). The ISAPP confirmed that the term ‘probiotic’ for food and food supplements should be used under certain conditions, including the administration of a minimum of 1 × 10^9^ CFU/day, a full genomic characterization of the probiotic strain and a history of safe use (Hill *et al*. 2014). Although a limited number of claims of health benefits of LAB have been approved, the probiotics market is thriving and is expected to grow further (Global Market Insight 2018). Probiotic strains are defined and potentially selected based on well-established criteria determined by the FAO and WHO (Araya *et al*. 2002). Strain identification, safety, stress tolerance and epithelial adherence capabilities are among the principal tests for screening probiotic strains (Pereira *et al*. 2018).

Owing to their food origin, some LAB species (mostly *Lactobacillus* and *Streptococcus* spp.) have a generally recognized as safe (GRAS) status according to the U.S. Food and Drug Administration (FDA) (https://www.accessdata.fda.gov/scripts/fdcc/?set=GRASNotices). In Europe, the concept of Qualified Presumption of Safety (QPS) was developed in 2007 by the EFSA to assist the safety assessment of microorganisms deliberately introduced into the food chain. The main difference between the GRAS and QPS concepts is that the former is generally limited to a specific application of a microorganism, while QPS refers to its generic safety in all possible uses. The QPS status evaluation is based on four points: taxonomy, scientific knowledge, the safety assessment (presence of virulence factors, production of toxins, antimicrobial resistance, reported cases of infection) and the expected end usage. When a species is included in QPS list, all the strains of that species will not need a full safety assessment (Sanders *et al*. 2010; Bourdichon, Laulund and Tenning 2019). Twenty-four *Lactobacillus* species, besides *Lactococcus lactis, Streptococcus thermophilus* and some *Leuconostoc* and *Pediococcus* species gained QPS status. Notably, no *Weissella* and *Enterococcus* spp. are included in this list (https://www.efsa.europa.eu/en/topics/topic/qualified-presumption-safety-qps). However, few cases of septicaemia induced by lactobacilli are also reported, but this typically occurs only in patients with pre-existing health problems, such as immunocompromised (O'Callaghan and O'Toole 2013).

Among LAB, at least ten species of *Lactobacillus* and *Lactococcus lactis* have been shown to exhibit probiotic properties, and their importance as health-promoting bacteria together with novel non-LAB species and strains has been recently reviewed (Douillard and de Vos 2019).

Therefore, an additional benefit of FFs is that they are natural sources of LAB, and as such, they can be regarded as ‘naturally potential’ functional foods. Regardless of the origin of the raw material, be it milk, vegetable or even meat, FFs can contain high loads of live LAB at the end of fermentation and in the final product. This does not apply simply to any FF. In fact, many foods obtained through fermentation do not contain live bacteria because they are inactivated by heat, as in the case of bakery products, or are physically removed, as in the case of alcoholic beverages (Rezac *et al*. 2018). Nevertheless, fermented milks, cheeses, fermented vegetables, meats, etc., do contain a considerable amount of live bacteria at consumption, which increases the number of microbes in the diet by up to 10 000-fold (Lang, Eisen and Zivkovic 2014). Diets rich in FFs offer remarkable microbial exposure in contrast with highly processed foods provided in societies with a high level of westernization and hygienic practices. Rezac *et al*. (2018) surveyed the amount of live LAB occurring in a variety of FFs at retail and found loads ranging between 10^5^ and 10^9^ CFU/g or ml, with dairy products containing the highest levels. Such high amounts of live LAB are therefore ingested with FFs and reach the human gastrointestinal tract (GIT). What happens after ingestion depends on the specific genetic and functional traits of the LAB strains and on their ability to resist the stress conditions to which they are exposed. High concentrations of pepsin and low pH (<3) are the principal barriers in the stomach, while bile and pancreatin are the typical adversities encountered in the small intestine. However, if they are able to endure to such stress factors, these bacteria can reach the colon and join the complex environment of the gut microbiome (see below).

### Fermented foods as source of microbial metabolites

Overall, the numerous enzymatic activities that can be carried out during food fermentation by LAB can change the biochemical composition of foods, releasing bioactive compounds that can provide health-promoting properties that the same matrix would not display without fermentation (Marco *et al*. 2017). Indeed, some LAB strains may exert health-promoting activity even if inactivated. The term ‘postbiotic’ was recently coined, indicating microbial metabolites or components of bacterial cell walls released in a matrix from which microbes are removed or inactivated and conferring health benefits when administered in sufficient amounts (Aguilar-Toalá *et al*. 2018). Such compounds include *β*-galactosidase for improved lactose digestion; conjugated linoleic acid, bioactive peptides and polyamines; and phenolic compound derivatives for oxidative stress improvement (Marco *et al*. 2017). LAB can also produce exopolysaccharides (EPSs) with potential cholesterol-lowering, antidiabetic, antioxidant, and immunomodulatory properties (Nampoothiri *et al*. 2017; Şanlier, Gökcen and Sezgin 2019). Several LAB produce B-group vitamins during fermentation and can effectively increase vitamin levels (LeBlanc *et al*. 2011). For example, *Lb. casei* KNE-1 was shown to synthetize thiamine (B_1_) and riboflavin (B_2_) in fermented milk drinks, while some strains of *S. thermophilus, Lb. delbrueckii* and *Lb. amylovorus* can be used to produce yogurts or fermented milks that are naturally rich in folate (B_9_), which is particularly important during pregnancy (Linares *et al*. 2017). In addition, some *Lc. lactis* strains can produce menaquinone (K_2_) in cheese and kefir (Walther *et al*. 2013). Other strains can produce neuroactive molecules, among which gamma-aminobutyric acid (GABA) is the most well studied. GABA acts as a neurotransmitter in mammals and performs additional functions, such as lowering blood pressure, relaxing muscles, and reducing psychological stress (Pessione and Cirrincione 2016; Şanlier, Gökcen and Sezgin 2019). The ability to produce GABA is recognized in several bacteria of gut origin (Wall *et al*. 2014), but fermented products rich in GABA have also been developed using specific strains of *Lb. casei, Lb. plantarum, S. thermophilus, Lb. brevis* and *Lc. lactis* as starters in fermented dairy products, legumes, cereals, and chocolate (Pessione and Cirrincione 2016; Linares *et al*. 2017).

Moreover, LAB can release biologically active peptides via proteolysis (Linares *et al*. 2017). The most well-studied peptides are antihypertensive peptides that can regulate blood pressure through inhibition of angiotensin-I-converting enzyme (ACE) and have been proposed as natural alternatives to antihypertensive drugs. ACE-inhibiting peptides are found mainly in fermented dairy products and fermented vegetables or legumes and are produced by several LAB used as starter cultures in FFs, including strains of *Lb. helveticus, Lb. casei, Lb. delbrueckii, Lb. plantarum, Lc. lactis*, and *S. thermophilus* (Shakerian *et al*. 2015; Li *et al*. 2017). In addition, peptides with different activities, such as anti-inflammatory, antioxidant, immunomodulatory, and antimicrobial activities, have also been identified in FFs (Pessione and Cirrincione 2016).

Another class of health-promoting molecules produced in FFs is conjugated fatty acids derived from bioconversion of linoleic acid (conjugated linoleic acid, CLA). CLA is naturally present in ruminant milk due to the activity of rumen bacteria, but the amount is by far sufficient to show some effects (Linares *et al*. 2017). Indeed, several LAB are known to produce CLA in milk products (*Lc. lactis, Lb. acidophilus, Lb. casei, Lb. plantarum, Lb. rhamnosus, Lb. delbrueckii*), and their use as starter or adjunct cultures may be a promising strategy for the production of enriched biofunctional foods (Linares *et al*. 2017).

Finally, LAB may reduce the presence of anti-nutritional compounds in FFs. An example is the phytase activity of some LAB. Phytic acid is present in several foods of vegetable origin, including cereals and legumes, and is considered an anti-nutrient substance since it can form complexes that chelate various minerals, thus reducing their bioavailability (Sharma *et al*. 2020). Phytase-producing LAB are able to hydrolyse phytates and release minerals. Different strains of *Lb. plantarum, Lb. amylovorus*, and *Lb. acidophilus* have been used for fermentation of sourdough from wheat, rye and oat; soy-based products; and beer and were able to reduce phytate concentrations in the fermented matrix (Sharma *et al*. 2020).

Besides exerting health-promoting activities and producing beneficial metabolites, some LAB strains are recognized as the main producers of biogenic amines (BA) in fermented foods from amino acids decarboxylation (Barbieri *et al*. 2019). The consumption of products containing high levels of BAs, depending on individual sensitivity or the concomitant assumption of specific drugs or ethanol, can cause headache, heart palpitations, vomiting, diarrhea and hypertensive crises (Barbieri *et al*. 2019). Moreover, several LAB strains have been shown to carry out genes responsible for antibiotic or antimicrobial resistance, that might be transferred to pathogens or GIT microbes (Campedelli *et al*. 2019). Therefore, in-depth and rigorous genomic characterization of food-related LAB strains is desirable to identify the presence of potentially dangerous activities.

### LAB prevalence and diversity in fermented foods

Fermentation has been traditionally used as an empirical method to improve food stability, and in recent years, it has been used to enhance the flavour, texture, and functional properties of food (Dimidi *et al*. 2019). LAB from several genera are commonly predominant in FFs, but other bacteria (e.g. propionibacteria and acetic acid bacteria), as well as fungi, also contribute to specific food fermentation processes.

**Figure 1.**
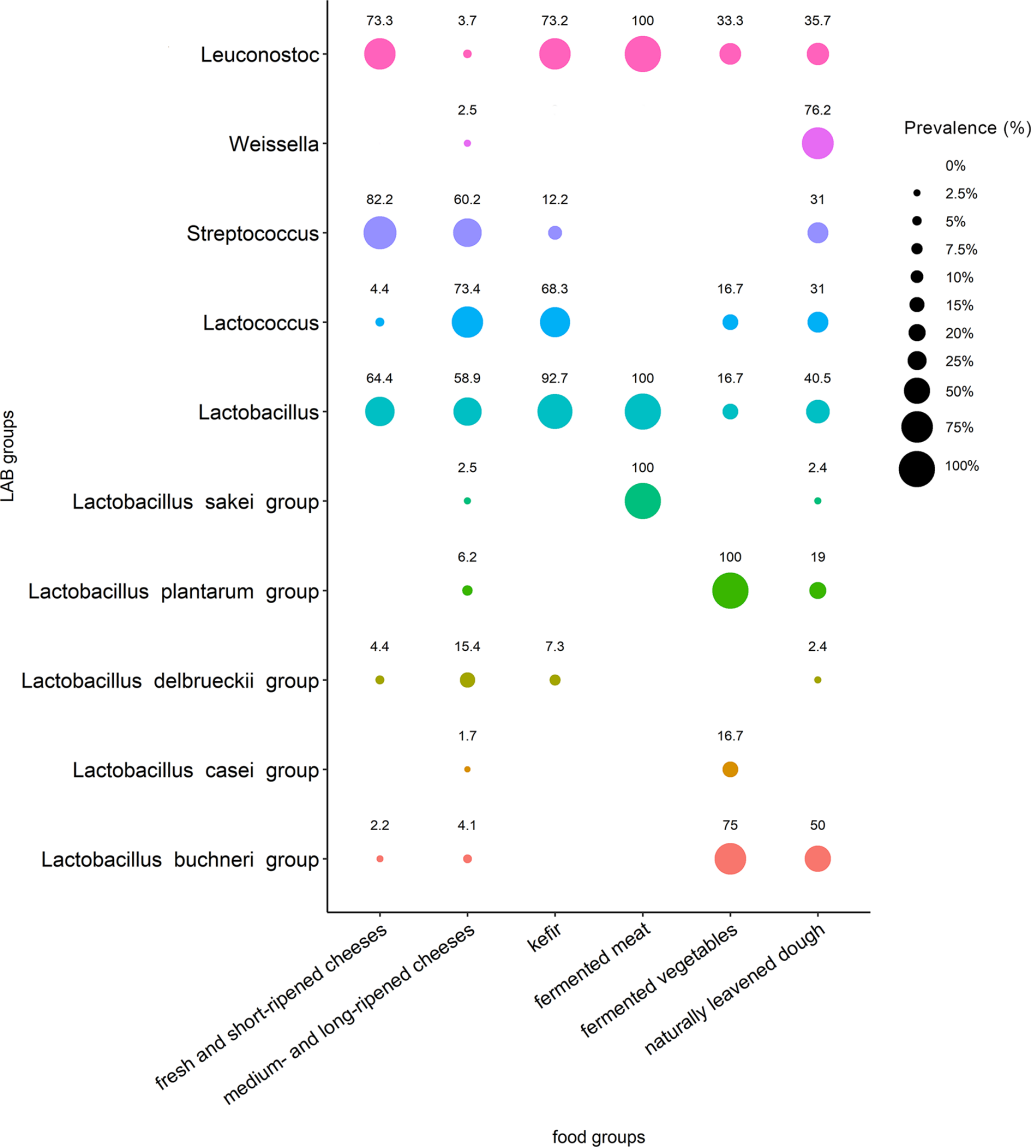
Bubble plot showing prevalence (% of samples) of LAB genera and species in different fermented foods, as obtained from 16S rRNA gene sequencing studies reported in FoodMicrobionet (Parente *et al*. 2019). A taxon was considered present if its relative abundance was > 0.5%. For lactobacilli, species were grouped into taxonomic groups, as reported by Salvetti *et al*. (2018).

*S. thermophilus, Lc. lactis, Leuconostoc* species, and several *Lactobacillus* species are the LAB most commonly found in FFs, either as naturally occurring bacteria or deliberately added as starter cultures. These species are among the most common commercially used bacteria, contributing to the production of yogurt, kefir, cheese and other dairy products; sauerkraut, kimchi and pickles; cured meat and fish; sourdough-based baked products; and many other traditional fermented foodstuffs around the world (Tamang *et al*. 2020). The main metabolic activity of interest for food production is the ability of these bacteria to carry out lactic acid fermentation, an anaerobic process that converts pyruvate molecules from glycolysis to lactic acid (homolactic fermentation) or lactic acid and other compounds, such as acetic acid, ethanol, and CO_2_ (heterolactic fermentation). These species can also activate several secondary metabolic processes that lead to the production of flavour compounds or typical textures. Combination of these metabolic processes leads to hundreds of different products, some of which are globally widespread, while many others are locally produced, often according to a traditional manufacturing practice (Chilton, Burton and Reid 2015; Tamang *et al*. 2020). Different food matrices can be considered specific ecological niches in which well-adapted LAB species finalize the fermentation process. In recent years, hundreds of studies have described microbial dynamics during the fermentation of different foodstuffs by high-throughput sequencing (HTS), extensively reviewed elsewhere (Ercolini 2013; De Filippis, Parente and Ercolini 2017, 2018b). Most of these studies are based on amplicon sequencing of taxonomically relevant genes and merely provide a survey of the microbial diversity occurring during food fermentation. Most of these studies have been collected in the FoodMicrobionet repository (http://www.foodmicrobionet.org; Parente *et al*. 2016; Parente *et al*. 2019). FoodMicrobionet contains data on microbial taxonomic composition from 44 HTS studies on food microbial ecology, including 29 datasets on food fermentation. To date, this repository includes a total of 2234 samples from food or food environments covering dairy, meat, fruits, vegetables, cereal-based foods, and ready-to-eat foods, with 806 samples of FF products. The samples are labelled according to the FoodEx classification (http://www.efsa.europa.eu/en/data/data-standardisation). Due to the availability of an app built with the Shiny R package, even inexperienced users can easily explore data, access external resources, filter samples based on multiple predefined criteria, aggregate samples and bacterial taxa, extract the taxonomic composition of specific groups of samples, and use them in comparative studies. We considered 806 samples spanning multiple FF matrices, extracted the prevalence of different LAB genera and species (collated in taxonomic groups, as defined by Salvetti *et al*. 2018), and grouped them according to the type of product or ripening time (Fig. 1). The niche specificity of *Lactobacillus* species is highlighted: *Lb. delbrueckii* group is prevalent mainly in dairy products, while the *Lb. plantarum* and *Lb. sakei* groups showed 100% prevalence in fermented vegetables and meat samples, respectively. *Lb. buchneri* group (including *Lb. buchneri, Lb. sanfranciscensis, Lb. brevis*) prevailed in sourdough, where *Lb. sanfranciscensis* is a well-known member of the microbial community (Ripari, Gänzle and Berardi 2016). Among other LAB genera, *Weissella* is found exclusively in naturally leavened sourdough, while *Streptococcus* and *Lactococcus* are found in cheeses and kefir. In addition, while most fresh and short-ripened cheeses contain thermophilic LAB, such as *Streptococcus*, high variability in LAB composition was found in ripened cheeses, in which mesophilic lactobacilli and *Lactococcus* are also present (Fig. 1). Some commonly consumed dairy products with a simple and defined microbiota structure (i.e. yogurt) are obviously not considered in Fig. 1 as they have not been studied by HTS approaches. LAB are often deliberately used for inoculation to start fermentation, as either selected commercial cultures or natural starters obtained according to a back-slopping procedure. Nevertheless, several artisanal products are fermented without the addition of starter microbes, but they arise from raw materials or from the facility environment, equipment and tool surfaces. Indeed, the food processing environment harbours a resident and complex microbiota that can be transferred to the product and represent a primary source of beneficial LAB (Montel *et al*. 2014; Stellato *et al*. 2015; Bokulich *et al*. 2016). Unfortunately, taxonomic identification at the species level is often not achievable with common amplicon-based HTS technologies, and many studies have reported genus-level identification (Fig. 1). This is a substantial limitation considering the wide species and subspecies diversity existing within LAB and the specific roles that these microbes can play during food production. This limitation may be overcome using a complex shotgun HTS approach, which remains underexploited in food-related microbiome studies. The use of metagenomics can be of invaluable importance for the identification of microbial genes and pathways leading to the production of metabolites associated with the typical sensorial profile of specific FFs, as well as for detecting potential health-related activities (De Filippis, Parente and Ercolini 2018b). In fact, in addition to producing lactic acid during fermentation, LAB confer important desirable properties to FFs. By degradation of carbohydrates, proteins and lipids, LAB can synthesize molecules positively associated with the flavours of FFs or modify the texture of some products by proteolysis, lipolysis or EPS production (Galle and Arendt 2014; Di Monaco *et al*. 2015; De Filippis *et al*. 2016; Gänzle and Ripari 2016; McAuliffe, Kilcawley and Stefanovic 2019). Nevertheless, it should be pointed out that identification by HTS does not imply that the microbes are alive at the moment of consumption, since these studies are usually based on DNA, which may be derived from dead or inactive cells. Viable counts of LAB in several FFs have also been reported (Rezac *et al*. 2018). It is estimated that between 10^8^ and 10^12^ CFU of bacteria may be ingested daily with the consumption of FFs (Derrien and van Hylckama Vlieg 2015). The quantities of *S. thermophilus* and *Lb. delbrueckii* in commercial yogurts and fermented milk vary from 10^4^ to 10^9^ CFU/ml, while the abundance of lactobacilli in cheeses ranges from 10^9^ to 10^3^ CFU/g, decreasing during ripening (Rezac *et al*. 2018). The levels of LAB in fermented sausages were reported to vary according to the origin, with fermented sausages from Europe showing higher counts than those from the US (<10^6^ vs 10^8^ CFU/g), which is probably associated with the more artisanal manufacturing process used for European products (Rezac *et al*. 2018). Therefore, the real amount of LAB ingested through a specific FF may also be extremely variable according to the geographical origin, manufacturing process (e.g. artisanal vs industrial; presence, length, and conditions of ripening; etc.), time and type of storage, and use of inactivation steps before consumption. For example, bread and other baked goods are usually cooked, while several fermented vegetables are pasteurized before commercialization to improve stability. However, the low levels of live microbes in the final product do not preclude a positive functional role. Indeed, several LAB may produce vitamins or other bioactive molecules *in situ* or inactivate anti-nutritional factors, thus exerting a positive health effect even if not alive at the time of consumption (Linares *et al*. 2017; see above).

## THE GUT MICROBIOME

The gut microbiome is among the most complex known microbial communities and among the most well studied. It comprises a very large variety of microbial strains belonging to species of bacteria, archaea, fungi and viruses that live in close relationship with the human host and whose combined genome harbours at least 100 times as many genes as the human genome (Bäckhed *et al*. 2005; Belkaid and Hand 2014). Members of the gut microbiome can influence host health through the production of a wide variety of beneficial or detrimental metabolites, and such molecules can be derived from both metabolic intermediates of the host and dietary precursors (Holmes *et al*. 2012; De Filippis *et al*. 2018a; Roager and Dragsted 2019). The most recent research advances have shown a high potential impact of the gut microbiome on the regulation of the equilibrium between health and disease, which is due to both the composition and functions of the microbiome. Complexity in composition is one of the main features of the gut microbiome, and microbial richness in terms of both species and genes has been linked to health (Cotillard *et al*. 2013; Le Chatelier *et al*. 2013; Vangay *et al*. 2018). In addition to differences in microbial composition based on health, lifestyle and geography (Almeida *et al*. 2019; Nayfach *et al*. 2019; Pasolli *et al*. 2019), the gut microbiome is characterized by high inter-individual variability (Truong *et al*. 2017). However, a large proportion of bacterial species are present in each individual and likely constitute a resilient microbial community (Aguirre de Cárcer 2018). In light of these considerations, the possible role of probiotics appears even more challenging, as after overcoming the barriers presented by the stomach and small intestine, probiotics encounter an army of hundreds of different species and strains to compete with, which affects their chances of exerting their beneficial effects. While the host-specific microbial community can be considered a resident microbiome, the microbes that we ingest and that reach the colon can be regarded as a transient microbiome, the composition of which depends on the type of exposure and on the type of food in the case of food-borne microorganisms (Derrien and van Hylckama Vlieg 2015). Indeed, the gut microbiome is exposed daily to microbes from the external environment, which are mainly of food origin. Probiotic LAB strains can be part of such a transient community and are supposed to perform their activity during passage through the gut and in the presence of the other members of the gut community.

Currently, it remains unknown which fraction of the food microbiome is actively transferred to the intestine and what type of activity those strains can exert in such a complex ecosystem. In fact, there is little literature on the prevalence of LAB in FF consumers and non-consumers. In addition, the possibility that non-probiotic food-borne LAB can also be transferred to the gut to a certain extent is currently underexplored, as are the actual activities that LAB can carry out in the gut as a complementary transient microbial community.

### LAB in the human gut

LAB are also widespread in other nutrient-rich environments, among which the human host is of great interest due to the potential functional properties of these bacteria (Soomro, Masud and Anwaar 2002; Duar *et al*. 2017b). The human microbiome interacts continuously with microbes originating from external environments, including food-origin sources. Probiotic LAB species and strains can constitute a portion of this transient microbiome and perform their activities during their transition in the gut, in addition to non-probiotic LAB that can potentially be transferred into the gut to a certain extent. Despite the availability of abundant nutrients, LAB present in the gut have to deal with a challenging scenario that involves hundreds of different bacterial and non-bacterial species sharing the same habitat (Pessione 2012).

Despite the long-term efforts in characterizing LAB, characterization of the effective contribution of these bacteria to the human microbiome remains a major challenge, and contradictory results have been reported in the literature (Walter 2008; Pessione 2012; George *et al*. 2018). Going back a hundred years, seminal studies identified lactobacilli, which we focus on here due to their prevalence in the LAB literature, among the most prevalent and abundant microorganisms in the human gut (Tannock 1999). At that time, techniques to cultivate anaerobic organisms were not yet available, which likely led to overestimation of the more easily cultivable microbes such as lactobacilli, while most of the gut (anaerobic) microbes remained undetected, a problem that despite continuous technological advancements has not yet been fully resolved (Almeida *et al*. 2019; Nayfach *et al*. 2019; Pasolli *et al*. 2019). For a long time, lactobacilli were considered to be numerically relevant members of the microbiome (Walter 2008), but most of the research conducted in the last few decades found that these bacteria are subdominant and therefore represent instead a small fraction of the overall microbiome composition. When using total anaerobic culturing techniques, the amount of lactobacilli rarely exceeded 10^8^ CFU/g and accounted for an average of 10^6^ CFU/g of intestinal content (Mitsuoka 1992; Walter *et al*. 2001; Dal Bello *et al*. 2003; Walter 2008), which represented a small fraction (< 0.01%) of the total count assuming that the intestinal content can reach up to 10^12^ CFU/g (O'Hara and Shanahan 2006). However, cultivation-based approaches may also be affected by biases because while lactobacilli can be easily isolated from food, isolation from human stool samples is more difficult since bifidobacteria are much more abundant and share similar nutritional requirements (Quartieri *et al*. 2016). On the one hand, findings obtained from culture-based approaches were confirmed in multiple studies by culture-independent methods (Walter 2008), which included, for example, fluorescent *in situ* hybridization (FISH) in combination with fluorescence microscopy (Harmsen *et al*. 2002), quantitative real-time PCR (Rinttilä *et al*. 2004), and high-throughput analysis of 16S rRNA gene amplicon sequencing data (Suau *et al*. 1999; Hayashi, Sakamoto and Benno 2002; Hold *et al*. 2002; Eckburg *et al*. 2005). The FISH data showed a relative abundance for the *Lactobacillus*/*Enterococcus* group ranging from 0.01–1.8% (Flint 2006; Louis *et al*. 2007). However, the results of other studies differed from these ones, instead finding that LAB occurrence may not be negligible in the human gut. This was mainly driven by advancements in 16S rRNA and metagenomics methodologies (Quince *et al*. 2017b), which made it possible to obtain largely unbiased perspectives on the relative importance of LAB in the context of the other members of the gut microbiota (Heeney, Gareau and Marco 2018). Approximately 5% and 13% of the sequences from 16S rRNA libraries were attributed to lactobacilli by (Frank *et al*. 2007) and (Hayashi *et al*. 2005), respectively. Using 16S rRNA data, *Lactobacillus* were estimated to constitute on average 6% of the bacterial cells in the duodenum (Nistal *et al*. 2016) and 0.3% in the colon (Almonacid *et al*. 2017). A longitudinal metagenomic study surveyed lactobacilli in a single person at three timepoints and found 52 subdominant species, 80% of which were detected in a two-year timeframe (Rossi *et al*. 2016). These results suggested that a relevant LAB population may be harboured in the human gut, with consistent inter-individual variations that may be driven by multiple factors, with the most likely one represented by diet (David *et al*. 2014) and the ingestion of LAB-enriched foods. Notably, most of this literature was derived from studies on faecal material, while very little is known about the small intestine microbiome due to it being accessible via only invasive procedures (Derrien and van Hylckama Vlieg 2015; El Aidy, van den Bogert and Kleerebezem 2015; Stolaki *et al*. 2019). This may represent an overlooked scenario because consumption of a dose of 10^10^ bacterial cells may have a strong influence, at least temporarily, on the microbial composition of the small intestine, since the microbial density in this organ, ranging from 10^4^ to 10^8^ bacteria/ml, is much lower than that in the colon (Derrien and van Hylckama Vlieg 2015).

Along with quantification of the LAB community in the gut, of comparable or even greater importance is the discrimination between resident (defined as autochthonous) and transient (allochthonous) components. This task is not trivial since LAB are continuously administered in the human ecosystem through ingested food and therefore represent a rather peculiar microbial group (Walter 2008; Rossi *et al*. 2016). Notably, populations of allochthonous species may appear stable if introduced regularly into the habitat (Duar *et al*. 2017b). Seminal studies on this topic were well summarized by (Walter 2008). Pioneering studies (Lerche 1961; Reuter 1965; Mitsuoka 1969) found transient and resident lactobacilli strains in stool samples, with the latter ones identified as *Lb. crispatus, Lb. gasseri, Lb. reuteri, Lb. ruminis*, and *Lb. salivarius* (Mitsuoka 1992; Reuter 2001). Further studies showed that a large fraction of the LAB species found in the gut are probably allochthonous and do not form stable populations, along with other species that can be considered autochthonous members of the microbiome (Tannock, Munro and Harmsen 2000; Walter *et al*. 2001; Walter 2008). For example, *Lb. ruminis* and *Lb. salivarius* were found to be persistent in multiple subjects for more than 18 months (Tannock, Munro and Harmsen 2000). A list of 17 lactobacillus species typically found in the gut was reported by (Walter 2008), comprising *Lb. acidophilus, Lb. brevis, Lb. casei, Lb. crispatus, Lb. curvatus, Lb. delbrueckii, Lb. fermentum, Lb. gasseri, Lb. johnsonii, Lb. paracasei, Lb. plantarum, Lb. reuteri, Lb. rhamnosus, Lb. ruminis, Lb. sakei, Lb. salivarius*, and *Lb. vaginalis*, most of which were identified as allochthonous members. This list was similarly reported by (Vaughan *et al*. 2002 and O'Callaghan and O'Toole 2013) and integrated with other LAB species. Some studies have also verified the colonization abilities of specific LAB strains. As an example, two strains of *Lb. mucosae* and *Lb. reuteri* reached higher population levels and were recovered more frequently from faecal samples than a strain of *Lb. acidophilus* (Frese, Hutkins and Walter 2012). Other studies determined the extent to which a host's persistent gut microbiota influences niche permissivity to transient LAB (Zhang *et al*. 2016) and how invasion by transient LAB can perturb the stability of microbial ecosystems (Amor, Ratzke and Gore 2019).

The quantities of LAB species that are persistent in the gut may be larger than currently documented. In the aforementioned work (Rossi *et al*. 2016), more than 40 species were detected in a single person in a two-year timeframe, indicating the need to conduct more and much larger analyses in similar settings.

Some untargeted studies have shown variations in the proportions of LAB in the gut and found positive or negative correlations with disease or chronic conditions. Depletion of intestinal lactobacilli was frequently associated with disease. As summarized in (Heeney, Gareau and Marco 2018), *Lactobacillus* was depleted under conditions of type 1 diabetes (de Goffau *et al*. 2014; Alkanani *et al*. 2015), irritable bowel syndrome (Liu *et al*. 2017; Zhuang *et al*. 2017), multiple sclerosis (Chen *et al*. 2016), human immunodeficiency virus infection (Yang *et al*. 2016), and prenatal stress (Zijlmans *et al*. 2015). On the other hand, enrichment of lactobacilli was verified in conditions of Crohn's disease (Wang *et al*. 2014; Lewis *et al*. 2017) and rheumatoid arthritis (Zhang *et al*. 2015). Contradictory findings were reported for type 2 diabetes (Karlsson *et al*. 2013; Forslund *et al*. 2015) and obesity (F. S. Teixeira *et al*. 2013; Ignacio *et al*. 2016).

Notably, most of the previous research has been devoted to the characterization of lactobacilli, while less attention has been given to other LAB members (Van den Bogert *et al*. 2013; Mignolet *et al*. 2016). Additionally, there is a lack of research aimed at assessing the distribution of LAB in the global population. This gap may be bridged by taking advantage of the growing availability of HTS data, as we will show below.

## DATABASES AND COMPUTATIONAL TOOLS TO RETRIEVE GENOME-WIDE INFORMATION ON THE PREVALENCE AND FUNCTIONAL DIVERSITY OF LAB FROM DIFFERENT SOURCES



Food and microbiome research can take advantage of the continuous improvement in HTS technology, which has revolutionized the microbial ecology field in the last two decades (Goodwin, McPherson and McCombie 2016). The continuous decrease in sequencing cost has been associated with exponential growth in terms of the number, diversity, and complexity of the sequenced data. Large international consortia have been established to mainly characterize the human microbiome (Human Microbiome Project (Human Microbiome Project Consortium 2012) and MetaHIT (Qin *et al*. 2010)), along with other initiatives such as the Tara Oceans Program (Sunagawa *et al*. 2015), the MetaSUB Consortium (MetaSUB International Consortium 2016), and the Earth Microbiome Project (Thompson *et al*. 2017), while such large efforts are still lacking in the food microbiome field.

Currently, two main approaches can be adopted in the microbiome field. The 16S rRNA gene sequencing method profiles selected organisms or single marker genes (Hamady and Knight 2009). It is the most cost-effective method, and the main output is limited to the generation of taxonomic profiles, typically at the genus level. Complete pipelines have been developed and widely used (Schloss *et al*. 2009; Bolyen *et al*. 2019), in addition to additional newly proposed methods (Callahan *et al*. 2016). More advanced analyses include oligotyping to obtain species- or even strain-level resolution (Eren *et al*. 2013) and (rough) estimation of functional potentials (Langille *et al*. 2013). Different repositories with annotated reference sequences have been made available and continuously updated (Pruesse *et al*. 2007; McDonald *et al*. 2012; Cole *et al*. 2014; Yoon *et al*. 2017). In addition, curated databases dedicated to specific environments of interest have been developed, for both 16S rRNA gene sequences and whole microbial genomes. DAIRYdb (Meola *et al*. 2019) provides a manually curated repository of 10,290 full-length 16S rRNA gene sequences from prokaryotes tailored for dairy product analyses. In addition, Almeida *et al*. (2014) developed a curated genome catalogue of 137 microbial species isolated from dairy products. Higher resolution can be obtained by acquiring the entire genomic content of a sample through (shotgun) metagenomic sequencing (Quince *et al*. 2017b). The large compendium of tools developed for this technique can be grouped into two main approaches, i.e. mapping-based profiling and *de novo* assembly. Species-level taxonomic profiles can be generated by adopting different mapping-based methods (Sunagawa *et al*. 2013; Wood and Salzberg 2014; Truong *et al*. 2015). Additional tools have also been developed to reduce errors by taking advantage of environmental and domain-specific information, which is however a quite overlooked research topic. This is the case of the methodology developed in (Seol *et al*. 2019) and aimed at reducing errors in terms of false positive rate for the specific identification of LAB and probiotic species.

Recently, attention has also been given to methodologies for metagenomic analysis with strain-level resolution. Different techniques have been proposed and are mainly based on the detection of single-nucleotide variants (SNVs) in the core genes (Costea *et al*. 2017; Truong *et al*. 2017), the identification of unique combinations of genes in the pangenome of a species (Scholz *et al*. 2016), or the use of a combination of these methods (Nayfach *et al*. 2016). Attempts have also been devoted to resolving multiple strains of the same species in a single sample (Quince *et al*. 2017a), although this remains an unresolved challenge along with the profiling of low-abundance non-dominant strains (Segata 2018). In addition to providing information on taxonomic composition, metagenomics can also be used for functional profiling (Franzosa *et al*. 2018). Complementary to mapping-based approaches is *de novo* metagenomic assembly. This method aims to provide (draft) genomes (defined as metagenome-assembled genomes, MAGs) of the microbial members present in samples. It can be used to expand the set of genomes of known and already studied species, but at the same time, due to its reference-free nature, provides the possibility to identify and characterize unknown members of the microbiome. Notably, MAGs can be integrated with genomes reconstructed from isolates and post-processed with a myriad of procedures based on comparative genomics that are quite standard for genomes from isolates. The idea of obtaining genomes directly from metagenomes is not new (Allen and Banfield 2005); however, this method was rarely applied until a few years ago due to computational challenges that have been addressed only recently. First, raw reads are assembled into contigs, with metaSPAdes (Nurk *et al*. 2017) and MEGAHIT (Li *et al*. 2015) representing the two most widely used tools. Then, the contigs are grouped into (draft) genomes through binning, with the popular tools represented by CONCOCT (Alneberg *et al*. 2014), MetaBAT2 (Kang *et al*. 2019), and DAS Tool (Sieber *et al*. 2018). Finally, only genomes of sufficient quality (usually evaluated in terms of completeness and contamination through tools such as CheckM (Parks *et al*. 2015) and BUSCO (Simão *et al*. 2015)) are retained and constitute the final set of genomes. Different papers devoted to the reconstruction and characterization of MAGs from large-scale scenarios have been recently published. Of great relevance is the characterization of the human microbiome (Almeida *et al*. 2019; Nayfach *et al*. 2019; Pasolli *et al*. 2019) along with the microbiomes, for examples, from the rumen (Stewart et al. 2018, 2019), non-human primates (Manara *et al*. 2019), and multiple other environments (Parks *et al*. 2017). However, similar efforts in the food microbiome field are still lacking.

The growing number of publicly available microbiome datasets enables hypothesis testing for environmental niches as well as meta-analyses across multiple studies. However, different factors prevent the research community from taking full advantage of these resources. These barriers include the need for substantial investment of time, computational resources and specialized bioinformatic expertise as well as inconsistencies in annotation and formatting between individual studies. To overcome these issues, in the last few years, several efforts have been devoted to the creation of resources and databases for the release of different types of microbiome data, which represent invaluable resources that allow the community to integrate newly acquired data with existing data. Comprehensive resources for both 16S rRNA and metagenomic data are represented by MGnify (Mitchell *et al*. 2020) and QIITA (Gonzalez *et al*. 2018). These resources integrate both the deposition of sequence data and distribution of products derived from multiple post-processing pipelines. Currently, MGnify (formerly EBI Metagenomics) integrates 214,977 samples spanning 3685 studies and is associated with six main biomes, i.e. the aquatic, food production, human, plant, soil, and wastewater biomes. While more than 40% of the samples are associated with the human microbiome, only a tiny fraction (< 1%) is related to food production systems. QIITA includes an even larger number of samples (i.e. 232,651 public and 137,644 private samples), although this resource is much more focused on 16S rRNA data. Additionally, in this case, very few public studies are associated with food.

Other resources of smaller size have also recently been made available and are focused on the collection, curation, and processing of samples derived from specific biomes and data types. A representative example of a resource focused on 16S rRNA data is FoodMicrobionet (Parente *et al*. 2019), already introduced above, which aims to retrieve and combine information specifically from food bacterial communities. Another interesting platform, although not specifically food focused, is Integrated Microbial Next Generation Sequencing (IMNGS (Lagkouvardos *et al*. 2016)). All prokaryotic 16S rRNA datasets available in Sequence Read Archive (SRA), which is the major database with permanent storage and public access to DNA sequencing data (Kodama *et al*. 2012), are systematically and uniformly screened and processed to build sample-specific sequence databases and OTU-based profiles. This integrative sequence resource can be queried by users through a web interface. It also offers a complete workflow for analysis of the user's own datasets for the sake of comparison with existing data. Other databases specifically focused on the human microbiome are represented by MicrobiomeHD (Duvallet *et al*. 2017) and HMP16SData (Schiffer *et al*. 2019). MicrobiomeHD includes 28 datasets from previously published case-control studies on the gut microbiome. OTU tables with associated taxonomic information and metadata for each sample can be easily downloaded. HMP16SData is a Bioconductor (Huber *et al*. 2015) package that provides count data for both 16S rRNA variable regions, integrated with phylogeny, taxonomy, and public participant data of the Human Microbiome Project (HMP). This is a good example in which, by removing the hurdles of data access and management, researchers with only basic R skills can analyse HMP data in a quick and simple way (Human Microbiome Project Consortium 2012).

Similar efforts to build databases for the human microbiome have also been conducted for shotgun metagenomics data. One example is the curatedMetagenomicData package (Pasolli *et al*. 2017), which currently includes more than 10,000 metagenomes from approximately 50 studies. This tool provides uniformly processed microbiome data, including bacterial, fungal, archaeal, and viral taxonomic abundances, in addition to quantitative metabolic functional profiles and standardized per-participant metadata. As in the case for HMP16SData, the data resources are accessible to users with minimal bioinformatic knowledge, and integration with the R/Bioconductor environment allows flexibility for researchers to perform novel analyses and methodological development and for integration of resources. Other similar resources that have been developed subsequently are Microbiome Learning Repo (ML Repo (Vangay, Hillmann and Knights 2019)) and Data Repository for Gut Microbiota (GMRepo (Wu *et al*. 2020)). ML Repo is a public, web-based repository of 33 curated classification and regression tasks from 15 already published datasets. GMRepo contains 58,903 human gut samples (17,618 from metagenomics and 41,285 from 16S rRNA data) spanning 253 datasets associated with 92 main phenotypes. In this case, the collected samples are organized according to their associated phenotypes. This tool is equipped with a graphical query builder, enabling users to make customized, complex and biologically relevant queries to obtain relevant information that is easy to access. Although such resources are related to the human microbiome in wide terms, at the same time they can be of interest for researchers interested in characterizing LAB in the human gut. Example of database developed for different biomes is the TerrestrialMetagenomeDB for terrestrial metagenomes (Corrêa *et al*. 2020), while similar products have not yet been developed for metagenomic studies from food microbiomes.

## AVAILABILITY OF LAB GENOMES IN FOOD AND THE GUT FOR COMPARATIVE STUDIES



Along with the availability of databases to improve the accessibility to raw sequences and their integration with metadata information, access to genome assemblies is also of great relevance. The benchmark in this context is the NCBI Assembly database (Kitts *et al*. 2016), which provides stable accessioning and data tracking for genome assembly data. Data can be found for different structures, such as sets of unordered contigs or scaffold sequences, bacterial genomes, or more complex structures, such as human genomes. A particular version of an assembly is identified unambiguously, and track changes are kept to identify genome updates. Along with the nucleotide sequences, this resource provides metadata such as assembly names, statistical reports of the assembly, and assembly update history. Users can easily download sequences and annotations through the NCBI Genomes FTP site.

**Figure 2.**
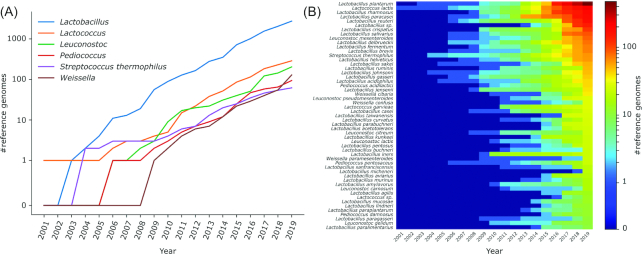
Number of LAB reference genomes in NCBI grouped at **(A)** genera and **(B)** species level. In (B), only species with at least 10 genomes deposited in NCBI on December 2019 are shown.

By searching the NCBI Assembly database with the keyword ‘prokaryotes’, we found 223,803 genomes. Filtering by taxonomy, we identified 3525 (1.6%) genomes associated with LAB species, namely, *Lactobacillus* (N = 2748), *Lactococcus* (N = 288), *Leuconostoc* (N = 204), *Pediococcus* (N = 96), *S. thermophilus* (N = 62), and *Weissella* (N = 129) (Fig. 2A and Supplementary Table S1). These genomes were associated with 257 taxa (Fig. 2B and Supplementary Table S2), with *Lb. plantarum* (N = 473), *Lc. lactis* (N = 223), *Lb. rhamnosus* (N = 191), *Lb. paracasei* (N = 183), and *Lb. reuteri* (N = 178), representing the most frequently occurring species. The first deposited strain was *Lc. lactis* subsp. *lactis* IL1403 in 2001 (Bolotin *et al*. 2001). The exponential growth of the number of available genomes is represented by the 80% increase in the number of deposited LAB genomes in the last 5 years (Fig. 2). This large number of public genomes represents a fundamental resource for mapping-based computational tools and for comparative genomics. For example, these genomes were used to propose a genome-based reclassification of the genus *Lactobacillus* (Wittouck, Wuyts and Lebeer 2019; Zheng *et al*. 2020) due to inconsistencies in the current taxonomy (Wuyts *et al*. 2017). The same approach was also recently applied to all publicly available bacterial and archaeal genomes (Parks *et al*. 2019).

**Figure 3.**
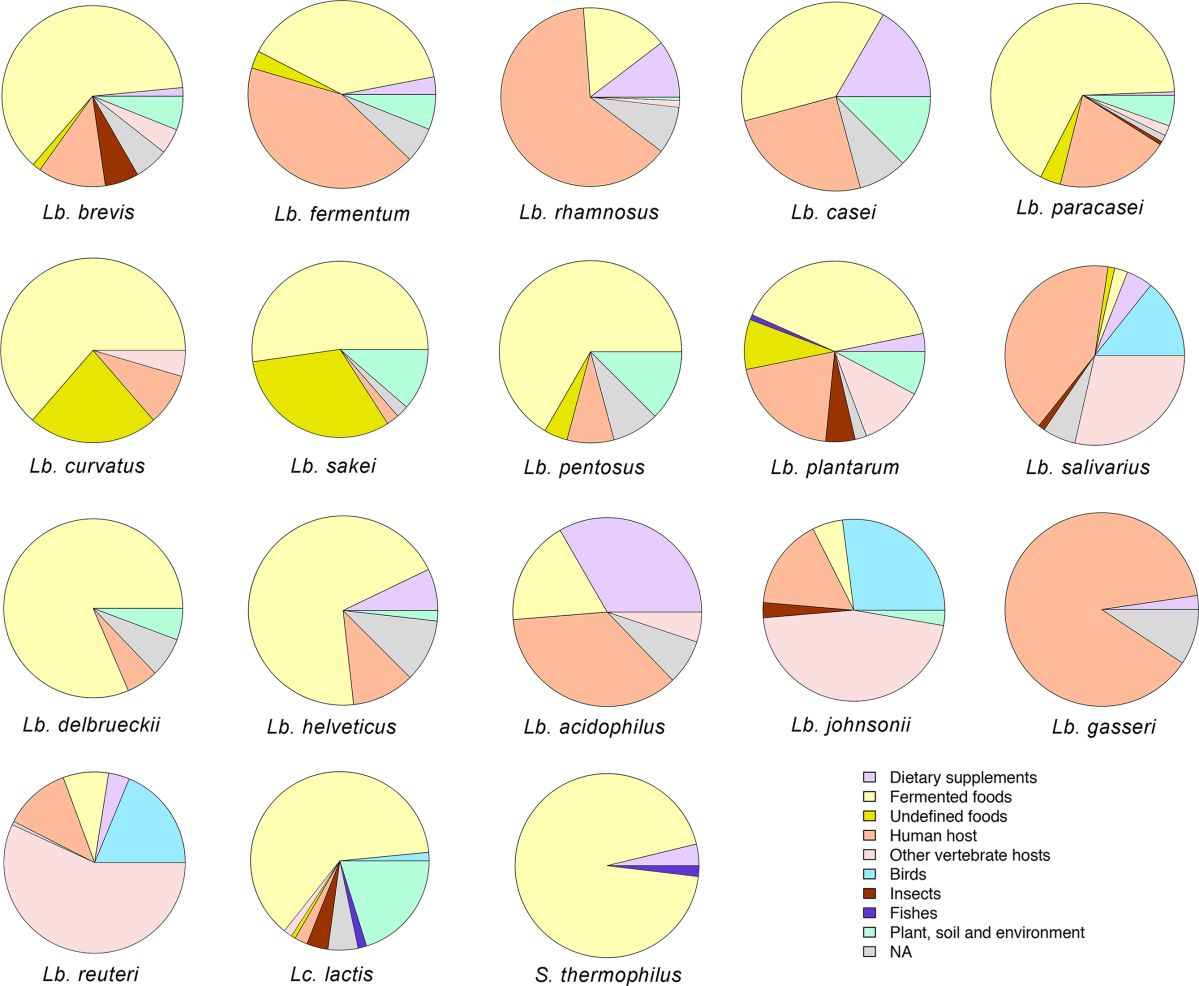
Pie charts showing isolation source for public genomes (available on NCBI in December 2019) of 18 selected LAB species, chosen for their importance in fermented foods and/or as probiotic. NA, Not Available. According to the taxonomy update proposed by Zheng *et al*. (2020), the names of the genera reported would change as follows: *Lb. brevis* = *Levilactobacillus brevis*; *Lb casei, Lb. paracasei* and *Lb. rhamnosus* = *Lacticaseibacillus* spp.; *Lb. fermentum* and *Lb. reuteri* = *Limosilactobacillus* spp.; *Lb. sakei* and *Lb. curvatus *= *Latilactobacillus* spp.; *Lb. plantarum* and *Lb. pentosus* = *Lactiplantibacillus* spp.; *Lb. salivarius* = *Ligilactobacillus*.

As reported above, LAB are widespread in natural environments. Considering the genomes from NCBI for 18 main LAB species frequently found in FFs or probiotic supplements, we summarized their source of isolation in Fig. 3 (as reported in NCBI or in the linked publications). FFs are the primary source of isolation for several LAB strains. In addition, host-adapted species can be identified. *Lb. gasseri*, a well-known probiotic species, was mainly isolated from the human infant gut, while *Lb. johnsonii, Lb. reuteri*, and *Lb. salivarius* were also retrieved from other animal hosts, both mammals and birds (Fig. 3). Nomadic lactobacilli (i.e. *Lb. casei, Lb. paracasei, Lb. plantarum*, and *Lb. rhamnosus*) were isolated from a variety of sources, including human, animal, and insect hosts, as well as soil and plant material (Fig. 3). Indeed, genomic comparisons highlighted that these species usually have a large genome size and a large number of coding sequences, allowing them to adapt and survive in a wide range of environments (Duar *et al*. 2017).

Along with the growing availability of reference genomes from isolated sequences, the number of MAGs retrieved from metagenomic datasets is continuously increasing, and these data can be combined for comparative genomics. To explore the availability of reconstructed genomes from LAB, we considered the large set of MAGs (N = 154,723) retrieved from 9428 human metagenomes that was clustered in 4930 species-level genome bins (SGBs) based on a 5% genetic diversity (Pasolli *et al*. 2019, 2020). Forty-nine of these SGBs belonged to LAB species, for a total of 830 reconstructed MAGs, grouped as follows: *Lactobacillus* (37 SGBs, 515 MAGs), *Lactococcus* (4 SGBs, 49 MAGs), *Leuconostoc* (3 SGBs, 7 MAGs), *Pediococcus* (2 SGBs, 5 MAGs), *S. thermophilus* (243 MAGs), and *Weissella* (2 SGBs, 11 MAGs) (Supplementary Table S3). These numbers are correlated with the occurrence of such species in the human gut, although the prevalence of low-abundance microbes is underestimated due to the technical impossibility of reconstructing MAGs from metagenomes in such cases. A large majority of the SGBs (44, 89.8%; for a total of 823 MAGs) represent at least partially known SGBs (kSGBs) that include one or more isolate genomes available in public databases. The most extensively reconstructed kSGBs were those of *S. thermophilus* (243 MAGs), *Lb. ruminis* (145 MAGs), *Lb. mucosae* (50 MAGs), *Lb. salivarius* (42 MAGs), and *Lb. rhamnosus* (32 MAGs). Only 7 MAGs spanning 5 SGBs were associated with unknown species (kSGBs), defined as SGBs lacking any publicly available genomes from isolate sequencing, which suggests the rarity of as-yet-uncharacterized LAB species in the human microbiome. Notably, reference genomes from human samples were almost entirely absent in the case of species with high prevalence in the gut, such as *S. thermophilus* and *Lc. lactis* (Fig. 3). Indeed, more than 90% of the *S. thermophilus* genomes were derived from FFs (mostly dairy products), while higher heterogeneity was observed for *Lc. lactis*, which was also found in insects, birds, fish and plant material (Fig. 3). Therefore, integrating isolated genomes with MAGs from large-scale metagenomic datasets can help overcome the lack of genomes from human hosts and represents an actual opportunity to advance the field through comparative genomic analyses of LAB, extensively taking into account different populations and environments of origin.

## PROBIOTIC TRAITS OF LAB THAT MAKE THEM RELEVANT FOR THE HUMAN GUT AND THEIR PREVALENCE ACROSS GENOMES



Several LAB species are GRAS, due to their centuries-long history of use and human consumption in FFs, and therefore include most of the probiotic species that are currently available on the market. This has boosted the search for and characterization of novel LAB strains with potential applications as probiotics. Indeed, a Scopus search for ‘probiotic’ and ‘Lactic Acid Bacteria’ returned approximately 4900 documents (December 2019).

The widespread genome sequencing efforts of recent years have led to the availability of hundreds of LAB genomes (Fig. 2), and the new term ‘probiogenomics’ was coined in 2009 (Ventura *et al*. 2009), describing a discipline aimed at exploring the evolutionary history of commensal and probiotic bacteria and highlighting the genetic bases of their health-promoting activities.

**Figure 4.**
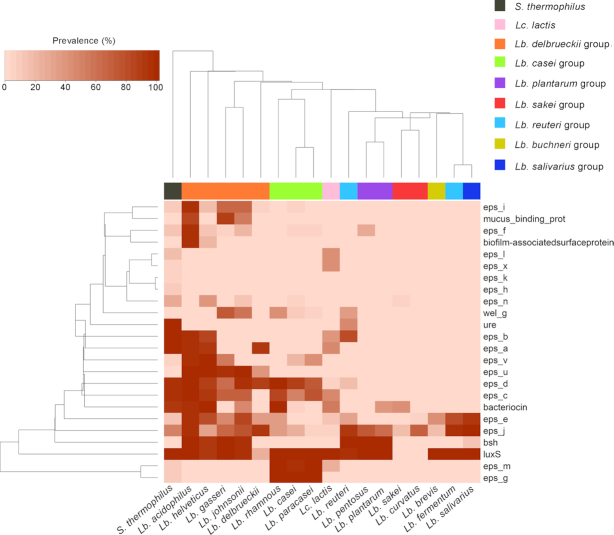
Heat plot showing prevalence of genes involved in resistance to the GIT passage and engraftment in the gut in public genomes (available on NCBI in December 2019) of 18 selected LAB species, chosen for their importance in fermented foods and/or as probiotic. *Eps*, genes involved in exopolysaccharides production; *ure*, urease; *bsh*, bile-salt hydrolase; *luxS*, S-ribosylhomocysteine lyase. A list of the genes included and their NCBI accession numbers are reported in Supplementary Table S4. Color bar indicates assignment to the different taxonomic groups, as reported by Salvetti *et al*. (2018). For the taxonomy update proposed by Zheng *et al*. (2020), see Figure 3 legend.

The first desirable feature in a probiotic strain is the ability to survive during passage through the GIT. For the scope of this review, we searched the publicly available genomes of 18 LAB species for 24 genes considered important for the capacity to resist the GIT, adhere to colonic cells, and colonize the intestine and that may be related to the ability of some LAB species commonly found in foods or supplements to reach the GIT and persist in the gut microbiome. A list of the genes and the relevant accession numbers is provided in Supplementary Table S4. Fig. 4 reports the prevalence of these genes in the genomes of 18 LAB species. The genes were predicted using Prokka (Seemann 2014) and were mapped using BlastN against a database containing the genes of interest. A gene was considered present if matched with an identity > 90% over a minimum length > 50%.

EPS production is known to protect microbial strains from acid and bile stress. EPSs are high-molecular-weight sugar polymers secreted by microorganisms into the surrounding environment. According to the chemical composition, two types of EPS, homopolysaccharides (HoPSs) and heteropolysaccharides (HePSs), are synthesized by LAB (Zannini *et al*. 2016). HoPSs are polymers of glucose or fructose, and depending on the type of molecular linkage, these polymers can be α-glucans, β-glucans, or β-fructans, while HePSs comprise two or more different monosaccharide units, mainly D-glucose, D-galactose, and L-rhamnose. Among food-borne LAB, EPS producers have been described in the genera *Streptococcus, Leuconostoc, Lactococcus, Pediococcus, Oenococcus, Lactobacillus* and *Weissella* (Zannini *et al*. 2016). In addition to protecting microorganisms from the GIT environment, some EPSs have been reported to exhibit immunomodulatory and anti-inflammatory properties (Castro-Bravo, Wells and Margolles 2018). The EPS gene cluster in LAB may include several glycosyltransferases, polysaccharide polymerases, a tyrosine kinase (*epsC*) and its modulator (*epsB*), a transcriptional regulator (*epsA*) and a phosphotyrosine phosphatase (*epsD*), which are differently distributed among different species (Deo, Davray and Kulkarni 2019). These genes are broadly spread in the genomes of the *S. thermophilus, Lc. lactis, Lb. casei*, and *Lb. delbrueckii* groups, in which many of them have been identified, but are less frequent in the *Lb. reuteri, Lb. brevis, Lb. fermentum*, and *Lb. curvatus* groups (Fig. 4). Several LAB strains with probiotic activities have developed mechanisms to counteract the hostile environment of the GIT, with low pH and the presence of bile acids. Urease activity is one of the mechanisms of defence against acid stress, degrading urea and producing ammonia, which increase the pH in the environment surrounding the microbial cell (Mora and Arioli 2014). Urease was present in the genomes of almost all *S. thermophilus* strains sequenced but was not detected in public genomes of other LAB species, except for approximately 47% of *Lb. reuteri* genomes (Fig. 4). In contrast, bile salt hydrolase (*bsh*) is present in > 90% of the genomes from *Lb. reuteri* and species within the *Lb. plantarum* and *Lb. delbrueckii* groups (Fig. 4). Bile salt hydrolase activity is considered desirable in probiotic strains since it allows the hydrolysis of conjugated bile salts, increasing the possibility of survival of the strain in the GIT (Begley *et al*. 2006).

Although several probiotic strains can exert a positive health effect without colonization of the GIT, adhesion to intestinal epithelial cells is one of the most commonly screened characteristics during preliminary probiotic characterization, as this property is essential for the competition of the probiotic strain with pathogens for resources and space (Papadimitriou *et al*. 2015). Colonic epithelial cells are covered by a layer of mucin, a large glycoprotein. Binding to colonic mucin by probiotic bacteria is achieved via a mucus-binding protein (*Mub*). In addition, the ability of bacterial cells to self-aggregate and form biofilms is considered to influence epithelial adhesion (Papadimitriou *et al*. 2015; Sanders *et al*. 2018). The *Mub* gene was found in 60–90% of the published genomes of *Lb. johnsonii, Lb. gasseri*, and *Lb. acidophilus* but was absent in the other LAB species screened, including phylogenetically related species such as *Lb. delbrueckii* and *Lb. helveticus*, all belonging to the same taxonomic group. In addition, approximately 94% of the *Lb. acidophilus* genomes contained a biofilm-associated surface protein. This highlights the high potential of these species to colonize the colonic environment and persist after ingestion. Indeed, these species include most of the commercially available probiotic strains.

Other interesting features that are potentially important for probiotic LAB are the presence of attachment factors, such as fimbriae and pili, and the production of antimicrobial compounds, such as acids, hydrogen peroxide or bacteriocins, which may enhance the ability of the bacteria to compete against other intestinal microbes and could potentially inhibit pathogens (Dobson *et al*. 2012; Sanders *et al*. 2019). Bacteriocins are small peptides synthetized ribosomally by a wide range of bacteria and archaea that exert antimicrobial activity against other taxa, either of the same species as the producer or across genera and against which the producer develops specific immunity-related mechanisms. Bacteriocins are a heterogeneous class of peptides with different structures, sizes, types of activity, immunity-related mechanisms, and target cell receptors (Dobson *et al*. 2012; Chikindas *et al*. 2018). More than 90% of the genomes of *S. thermophilus, Lb. acidophilus*, and *Lb. helveticus* showed genes involved in bacteriocin production, while these genes were present in approximately 40–50% of *Lb. sakei, Lb. plantarum, Lb. johnsonii*, and *Lc. lactis* genomes (Fig. 4). However, the importance of bacteriocin production in probiotic bacteria remains controversial (Dobson *et al*. 2012). Many bacteriocin-producing microorganisms can inhibit pathogens *in vitro* (Le Blay *et al*. 2007), but results regarding *in vivo* efficacy are scarce and often do not match the *in vitro* activity. For example, the antimicrobial peptide lacticin 3147 produced by an *Lc. lactis* strain was effective against *Listeria monocytogenes in vitro* but failed in a mouse model (Dobson *et al*. 2011). The same result was reported for a strain of *Pediococcus acidilactici*: a corresponding effect was not observed *in vivo* despite its activity in the reduction of *L. monocytogenes* viability by 3 logs *in vitro* (Dabour *et al*. 2009). In contrast, some LAB strains are able to synthetize bacteriocins *in vivo*, showing direct antagonistic activity against pathogens. For example, Corr *et al*. (2007) demonstrated that oral gavage of *Lb. salivarius* UCC118 into mice protected them from *L. monocytogenes* infection. However, we should note that bacteriocins usually show a limited spectrum of action against target bacteria that are phylogenetically close to the producer. Therefore, bacteriocins produced by LAB are usually active against only Gram-positive bacteria.

## HEALTH-RELATED ACTIVITIES OF LAB: EVIDENCE FROM *IN VIVO* TRIALS



Several studies have reported the health-related functional properties of probiotic LAB. Anti-inflammatory and immunomodulatory effects have been proposed for some LAB strains. For example, a strain of *Lb. plantarum* was suggested to play an anti-inflammatory role due to a specific structure of its teichoic acids (Grangette *et al*. 2005), while this role was attributed to EPS (Górska *et al*. 2016), pili (Lebeer *et al*. 2012) and S-layer proteins (Konstantinov *et al*. 2008) in strains of *Lb. plantarum, Lb. rhamnosus*, and *Lb. acidophilus*, respectively. A strain of *Lb. salivarius* was able to reduce inflammation and exert a preventive effect on colitis development in mice (Daniel *et al*. 2006), and the administration of a heat-killed strain of *Lb. plantarum* ameliorated inflammation and fibrosis in obese rats (Uchinaka *et al*. 2018). In addition, contact of the growth supernatant of one strain of *S. thermophilus* with immune cells reduced the release of the inflammatory marker TNF-alpha (Ménard *et al*. 2004).

The role of LAB in obesity and the related metabolic syndrome remains controversial, and contrasting results have been reported in the literature. The abundance of lactobacilli was found to be higher in obese subjects than in anorexic subjects (Ley *et al*. 2005) and in type 2 diabetes patients (Larsen *et al*. 2010; Karlsson *et al*. 2013). In addition, Drissi *et al*. (2014) suggested that *Lactobacillus* species might be differently associated with weight gain or loss; moreover, an enhanced potential for glycolysis, fat digestion, and oxidative stress response in weight gain-associated strains was highlighted when comparing the genomes of the two groups. However, several studies on mouse models have shown that the LAB consumption improved glucose metabolism and hepatic inflammation associated with a high-fat diet (Alard *et al*. 2016; Park *et al*. 2017). A similar result was also observed in human clinical trials. Moroti *et al*. (2012) hypothesized an effect of daily consumption of *Lb. acidophilus* and *B. bifidum* strains in reducing glycaemia and cholesterol levels, while Kobyliak *et al*. (2018b) concluded that supplementation with a multistrain probiotic (containing strains of *Lactobacillus, Lactococcus, Bifidobacterium, Acetobacter, Propionibacterium*) reduced insulin resistance in type 2 diabetes patients. All these contrasting results highlighted that the role of LAB cannot be generalized and that different species and strains can have a specific effect. Moreover, the overall response is also likely influenced by inter-individual variability.

Most of our knowledge is based on *in vitro* experiments or animal trials, while the best option to ascertain the possible health benefits of a microbial strain is human randomized controlled trials (Hill *et al*. 2014; De Filippis *et al*. 2018a). We surveyed the available literature regarding the effect of dietary intervention with probiotic LAB by searching the Scopus database for documents (only articles written in English) containing the words ‘probiotic’ OR ‘lactic acid bacteria’ AND ‘clinical trial’ OR ‘intervention’ OR ‘treatment’ in the abstract, title or key words. Animal trials were excluded, as well as review articles and studies in which probiotic strains not belonging to the LAB group were exclusively tested. This search identified a total of 95 studies, which are reviewed and reported in Table 1. Probiotic consumption has been extensively tested in the literature in either healthy or diseased populations (Table 1). Most of the studies used multistrain probiotic products, containing 1 to 10 different strains, with high heterogeneity in the amount ingested, ranging mostly from approximately 10^7^ to 10^11^ cells/day, in single or multiple doses. When multistrain formulations were used, LAB were often administered together with *Bifidobacterium* spp. strains. In addition, mixed preparations of probiotic strains and prebiotic fibre are often used (symbiotic). Supplementation with probiotic LAB has been proposed for the treatment or improvement of symptoms of several types of diseases, including inflammatory bowel diseases, allergies and intolerance, diabetes and metabolic syndrome, stress and mental disorders, and infant colic. It is important to note that several studies did not report the name of the specific strain(s) used, the viable counts and the number of cells ingested, making comparison across studies impossible. Indeed, many of the probiotic activities were strain specific. For example, two different strains of *Lb. acidophilus* (LA-5 and NCFM) were tested at a similar dose for a possible role against allergic diseases (asthma and atopic dermatitis, respectively), and only *Lb. acidophilus* LA-5 was shown to be effective (Table 1).

**Table 1.** Human trials involving probiotic LAB administration.

Probiotic species/strains	Quantity/microbial loads ingested	Participants	Placebo control group	Study design*	Participant characteristics	Length of intervention	Wash out	Gut microbiome analysis method	Variation in gut microbiome detected	Health outcome targeted	Health outcome achieved*	Reference
*B. animalis* sub*. lactis* CNCM I-2494*, Lb. delbrueckii* sub. *bulgaricus* CNCM I-1632 and CNCM I-1519*, S. thermophilus* CNCM I-1630*, Lc. lactis* sub. *lactis* CNCM I-1631	Counts and daily amount assumed not reported	50	Yes	Double-blind, randomized, placebo-controlled trial	Male (36%), adults (35 ± 11), normal-weight	2 weeks	No	16S rRNA gene sequencing	None	IBS symptoms	No	Le Nevé *et al*. 2019
Bio-25–B. bifidum, Lb. rhamnosus, Lc. lactis, Lb. casei sub. casei, B. breve, S. thermophilus, B. longum sub. longum, Lb. casei sub. paracasei, Lb. plantarum, B. longum sub. infantis; strains not reported	2 pills/day, 2.5 × 10^10^ CFU/pill	8	No	Not reported	Male (63%), adults (28 ± 2.3), normal-weight	4 weeks	No	16S rRNA gene and shotgun sequencing	Increase in *Akkermansia, Vagococcus, Enterococcus, Blautia*, and *Lactococcus*	Gut microbiota reconstruction upon antibiotic administration	No	Suez *et al*. 2018
Dicoflor60®—*Lb. rhamnosus* GG	2 capsules/day, 6 × 10^9^ CFU/capsule	45	No	Not reported	Male (84%), adults (47 ± 8), normal-weight	8 weeks	No	16S rRNA gene sequencing	Decrease in *Enterobacteriaceae*	Inflammatory status in HIV patients	Yes	Arnbjerg *et al*. 2018
DUOLAC 7–*S. thermophilus* KCTC 11870BP, *Lb. plantarum* KCTC 10782BP, *Lb. acidophilus* KCTC 11906BP, *Lb. rhamnosus* KCTC 12202BP, *B. lactis* KCTC 11904BP*, B. longum* KCTC 12200BP*, B. breve* KCTC 12201BP	2 capsules/day, 5 × 10^9^ CFU/capsule	17	Yes	Double-blind, randomized, placebo-controlled trial	Male (0%), obese; age not reported	8 weeks	No	Real-time qPCR for specific targets	Increase in *B. breve, B. lactis* and *Lb. rhamnosus*	Endotoxemia in obese subjects	Yes	Lee *et al*. 2014
Ecologic® 825–*Lb. casei* W56, *Lb. acidophilus* W22, *Lb. paracasei* W20, *B. lactis* W51, *Lb. salivarius* W24, *Lc. lactis* W19, *B. lactis* W52, *Lb. plantarum* W62*, B. bifidum* W23	3 g/day, 7.5 × 10^6^ CFU/g	15	Yes	Double-blind, randomized, placebo-controlled trial	Male (50%), adults (26 ± 5), BMI not reported	4 weeks	No	None	-	Participants behaviour	Yes	Bagga *et al*. 2019
Ecologic® Barrier—*B. bifidum* W23*, B. lactis* W51*, B. lactis* W52, *Lb. acidophilus* W37*, Lb. brevis* W63*, Lb. casei* W56*, Lb. salivarius* W24*, Lc. lactis* W19, *Lc. lactis* W58	4 g/day, 2.510^9^ CFU/g	34	Yes	Placebo-controlled trial	Male (38%), adults (36 ± 12), normal-weight	8 weeks	No	16S rRNA gene sequencing	None	Depression	Yes	Chahwan *et al*. 2019
Ecologic® Barrier—see above	6 g/day, 2.5 × 10^9^ CFU/g	12	Yes	Double-blind, randomized, placebo-controlled trial	Male (92%), adults (61 ± 5), obese	24 weeks	No	16S rRNA gene sequencing	Increase in *Lb. brevis*	Glucose metabolism in type-2 diabetes patients	No	Horvath *et al*. 2019
Ecologic® Barrier—see above	2 g/day, 2.5 × 10^9^ CFU/g	29	Yes	Double-blind, randomized, placebo-controlled trial	Male (0%), adults (21 ± 1), normal-weight	4 weeks	No	None	-	Neurocognitive performance	Yes	Papalini *et al*. 2018
Ecologic® Barrier (see above)	2 g/day, 2.5 × 10^9^ CFU/g	20	Yes	Placebo-controlled trial	Male (25%), adults (20 ± 2), normal-weight	4 weeks	No	None	-	Depression	Yes	Steenbergen *et al*. 2015
Ecologic® Barrier (see above)	1 serving/day, 2.5 × 10^9^ or 1 × 10^10^ CFU/serving	30	Yes	Double-blind, randomized, placebo-controlled trial	Male (0%), adults (56 ± 7), obese	12 weeks	No	None	-	Iron metabolism in post-menopausa	Yes	Skrypnik *et al*. 2019
Enterolactis® Plus—*Lb. paracasei* DG	2 capsules/day, 2.4 × 10^10^ CFU/capsule	30	Yes	Double-blind, randomized, placebo-controlled, cross-over trial	Male (48%), adults (35 ± 11), normal-weight	4 weeks	Yes, 4 weeks between treatments	16S rRNA gene sequencing	Increase in Proteobacteria and *Coprococcus*; decrease in *Blautia*	None	-	Ferrario *et al*. 2014
Enterolactis® Plus—see above	Counts and daily amount assumed not reported	20	Yes	Double-blind, randomized, placebo-controlled, cross-over trial	Male (25%), adults (45 ± 13), BMI not reported	4 weeks	Yes, 4 weeks between treatments	16S rRNA gene sequencing	Increase in *Lactobacillus* and *Oscillospira*, decrease in *Ruminococcus*	IBS symptoms	Yes	Cremon *et al*. 2018
GoodBelly StraightShot—*Lb. plantarum* Lp299v	1 serving/day, 2 × 10^10^ CFU/serving	20	No	Not reported	Male (100%), adults (63 ± 7), obese	6 weeks	Yes, 4 weeks	16S rRNA gene sequencing	Increase in *Lactobacillus*	Coronary artery disease	Yes	Malik *et al*. 2018
Heat-killed *Lb. gasseri* CP2305	2 tablets/day, 1 × 10^10^ CFU/tablet	31	Yes	Double-blind, randomized, placebo-controlled trial	Male (68%), adults (25 ± 0.5), normal-weight	24 weeks	No	16S rRNA gene sequencing	Decrease in *Bifidobacterium, Streptococcus*	Stress and anxiety	Yes	Nishida *et al*. 2019
Heat-killed *Lb. paracasei* CBA L74	1 serving/day, 5.9 × 10^11^ CFU/serving	66	Yes	Double-blind, randomized, placebo-controlled trial	Male (53%), infants (33 ± 9 months)	12 weeks	No	None	-	Infection disease occurrence in children	Yes	Corsello *et al*. 2017
Heat-killed *Lb. paracasei* CBA L74	1 serving/day, 5.9 × 10^11^ CFU/serving	10	Yes	Double-blind, randomized, placebo-controlled trial	Male (80%), children (33 ± 9 months)	12 weeks	No	16S rRNA gene sequencing	Increase in *Lactobacillus, Faecalibacerium, Oscillospira*	None	-	Berni Canani *et al*. 2017
LacClean Gold-S®—*B. bifidum* KCTC 12199BP, *B. lactis* KCTC 11904BP, *B. longum* KCTC 12200BP, *Lb. acidophilus* KCTC 11906BP, *Lb. rhamnosus* KCTC 12202BP, *S. thermophilus* KCTC 11870BP	2 capsules/day, 5 × 10^9^ CFU/capsule	25	Yes	Double-blind, randomized, placebo-controlled trial	Male (44%), adults (46 ± 14), BMI not reported	4 weeks	No	Real-time qPCR for specific targets	Increase in *B. lactis, Lb. rhamnosus, S. thermophilus*	IBS symptoms	Yes	Yoon *et al*. 2014
*Lb. acidophilus* LA-5*, B. animalis* subsp*. lactis* BB-12	1 serving/day, 1 × 10^9^ CFU/serving	23	Yes	Double-blind, randomized, placebo-controlled trial	Male (53%), adults (52 ± 7), overweight	6 weeks	No	None	-	Glucose metabolism in type-2 diabetes patients	Yes	Bordalo Tonucci *et al*.^2015^
*Lb. acidophilus* LA-5*, Lb. rhamnosus* GG*, B. animalis* subsp. *lactis* BB-12	1 capsule/day, 8.75 × 10^9^ CFU/capsule	16	Yes	Double-blind, randomized, placebo-controlled three way cross-over trial	Male (50%), adults (43 ± 20), over-weight	1 week	Yes, 2 weeks between treatments	Real-time qPCR and 16S rRNA gene sequencing	Increase in *Bifidobacterium*	Asthma	Yes	McLoughlin *et al*. 2019
*Lb. acidophilus* NCFM OR *B. lactis* Bi-07	1 serving/day, 1 × 10^10^ CFU/serving	17	Yes	Double-blind, randomized, placebo-controlled trial	Infants (7–24 months); sex not reported	8 weeks	No	16S rRNA gene sequencing	None	Atopic dermatitis	No	Larsen *et al*. 2011
*Lb. acidophilus* PBS066*, Lb. reuteri* PBS072 OR *Lb. plantarum* PBS067*, Lb. rhamnosus* LRH020*, B. animalis* subsp. *lactis* BL050	1 serving/day, 5 × 10^9^ CFU/serving	50	Yes	Double-blind, randomized, placebo-controlled trial	Adults (37 ± 13), sex and BMI not reported	8 weeks	Yes, 4 weeks	None	-	IBS with constipation	Yes	Mezzasalma *et al*. 2016
*Lb. acidophilus, B. bifidum, B. lactis, B. longum;* strains not reported	1 serving/day, 1.5 × 10^9^ CFU/serving	27	Yes	Double-blind, randomized, placebo-controlled trial	Male (48%), adults (53 ± 6), BMI not reported	24 weeks	No	None	-	Metabolic syndrome in pre-diabetic patients	Yes	Kassaian *et al*. 2019
*Lb. acidophilus, B. longum*; strains not reported	2 capsules/day; counts not provided	37	Yes	Double-blind, randomized, placebo-controlled trial	Male (30%), adults (44 ± 15), normal-weight	4 weeks	No	Real-time qPCR for specific targets	None	IBS symptoms	Yes	Cui and Hu 2012
*Lb. acidophilus, Lb. casei, Lb. lactis, B. bifidum, B. longum, B. infantis;* strains not reported	2 sachets/day, 3 × 10^10^ CFU/sachet	68	Yes	Double-blind, randomized, placebo-controlled trial	Male (46%), adults (53 ± 9), normal-weight or overweight	12 weeks	No	Microbial counts	Increase in *Bifidobacterium* and *Lactobacillus*	Glucose metabolism in type-2 diabetes patients	Yes	Firouzi *et al*. 2017
*Lb. acidophilus, Lb. plantarum, Lb. fermentum, Lb. gasseri;* strains not reported	0.5 g/day, 5 × 10^10^ CFU/g	45	Yes	Double-blind, randomized, placebo-controlled trial	Male (0%), adults (30 ± 6), normal-weight	6 weeks	No	None	-	Glucose metabolism in women with gestational diabetes	No	Nabhani *et al*. 2018
*Lb. casei* 01	1 capsule/day, 1 × 10^8^ CFU/capsule	22	Yes	Double-blind, randomized, placebo-controlled trial	Male (0%), adults (41 ± 13), overweight	8 weeks	No	None	-	Inflammatory status in rheumatoid arthritis	Yes	Vaghef-Mehrabany *et al*. 2014
*Lb. casei* Lcr35®	2 capsules/day, 2 × 10^8^ CFU/capsule	42	No	Prospective, randomized, case-controlled study	Male (57%), children (2.3 ± 1.3), normal-weight	1 week	No	16S rRNA gene sequencing and plate counts	Increase in *Lachnospiraceae, Bacteroides, Ruminococcus* and decrease in *Enterobacteriaceae, Escherichia, Clostridium*	Diarrhea	Yes	Lai *et al*. 2019
*Lb. casei* LMG 101/37 *P*-17 504*, Lb. plantarum* CECT 4528*, B. animalis* sub. *lactis* Bi1 LMG *P*-17 502*, B. breve* Bbr8 LMG *P*-17 501, *B. breve* Bl10 LMG *P*-17 500	1 sachet/day, 10 × 10^9^ CFU/sachet	54	Yes	Double-blind, randomized, placebo-controlled trial	Male (11%), adults (43 ± 10), normal-weight	6 weeks	No	16S rRNA gene sequencing and plate counts	Increase in *Bifidobacterium, Staphylococcus* and lactic acid bacteria	Concomitant celiac disease and IBS	Yes	Francavilla *et al*. 2019
*Lb. casei* W56*, Lc. lactis* W19*, Lb. acidophilus* W22*, B. lactis* W52*, Lb. paracasei* W20*, Lb. plantarum* W62*, B. lactis* W51*, B. bifidum* W23*, Lb. salivarius* W24	1 sachet/day; counts not reported	20	No	Not reported	Male (55%), adults (77 ± 10), BMI not reported	4 weeks	No	Real-time qPCR for specific targets	Increase in *Faecalibacterium prausnitzii*	Alzheimer's disease	Yes	Leblhuber *et al*. 2018
*Lb. casei* Zhang	1 tablet/day, 1 × 10^10^ CFU/tablet	24	No	Open-label	Male (46%), adults (43 ± 17), normal-weight	4 weeks	No	16S rRNA gene sequencing	Positive correlation of *Lb. casei* with *Prevotella, Faecalibacterium, Propionibacterium, Bifidobacterium*	None	-	Zhang *et al*. 2014
*Lb. casei, Lb. acidophilus, Lb. rhamnosus, Lb. delbrueckii* subsp. *bulgaricus, B. breve, B. longum, S. thermophilus;* strains not reported	1 capsule/day, 1.5 × 10^10^ CFU/capsule	30	Yes	Double-blind, randomized, controlled trial	Male (7%), adults (42 ± 2), normal-weight	8 weeks	No	None	-	Thyroid function	Yes	Talebi *et al*. 2020
*Lb. casei, Lb. rhamnosus, S. thermophilus, B. breve, Lb. acidophilus, B. infantis, Lb. bulgaricus*; strains not reported	1 serving/day, counts not reported	25	Yes	Double-blind, randomized, placebo-controlled trial	Male (60%), infants (1.5 ± 0.8 months)	4 weeks	No	None	-	Crying time in infants with colic	Yes	Kianifar *et al*. 2014
*Lb. casei, Lb. rhamnosus, S. thermophilus, B. breve, Lb. acidophilus, B. longum, Lb delbrueckii* sub. *bulgaricus;* strains not reported	2 capsules/day, 2 × 10^8^ CFU/capsule	20	Yes	Parallel, triple-blind, randomized, controlled trial	Adults (57 ± 1.5), over-weight/obese, sex not reported,	12 weeks	No	None	-	Metabolic syndrome	Yes	Rabiei *et al*. 2019
*Lb. casei, Lb. rhamnosus, S. thermophilus, B. breve, Lb. acidophilus, B. longum, Lb. bulgaricus*; strains not reported	1 capsule/day, 2 × 10^8^ CFU/capsule	29	Yes	Randomized triple-masked controlled trial	Male (45%), children (11 ± 2), obese	4 weeks	No	Microbial counts	None	Obesity	No	Kelishadi *et al*. 2014
*Lb. gasseri* CECT5714*, Lb. coryniformis* CECT5711	1 serving/day, 2 × 10^9^ CFU/serving	15	Yes	Double-blind, randomized, placebo-controlled trial	Male (50%), adults (33 ± 10), BMI not reported	4 weeks	Yes, 2 weeks	None	-	Bowel habits	Yes	Olivares *et al*. 2006
*Lb. gasseri* KS-13*, B. bifidum* G9–1*, B. longum* MM2	2 capsules/day, 1.5 × 10^9^ CFU/capsule	16	Yes	Double-blind, randomized, placebo-controlled, cross-over trial	Adults (70 ± 1), sex and BMI not reported	3 weeks	Yes, 5 weeks between treatments	Real-time qPCR for specific targets and 16S rRNA gene sequencing	Increase in *Faecalibacterium prausnitzii*	Inflammatory status in older people	Yes	Spaiser *et al*. 2015
*Lb. helveticus* Bar13, *B. longum* Bar33	1 serving/day, 1 × 10^9^ CFU/serving	16	Yes	Double-blind, randomized, placebo-controlled trial	Male (41%), adults (76 ± 10), BMI not reported	4 weeks	No	Phylogenetic microarray	Decrease in opportunistic pathogens (*Clostridium difficile, Cl. perfringens, Enterococcus faecium*)	None	-	Rampelli *et al*. 2013
*Lb. helveticus* R0052	1 sachet/day, 3 × 10^9^ CFU/sachet	23	Yes	Randomized, double-blind, placebo- controlled trial	Infants (3–12 months), sex and BMI not reported	8 weeks	No	16S rRNA gene sequencing	Increase in Proteobacteria, decrease in Firmicutes	Inflammatory status	No	De Andrés *et al*. 2018
*Lb. helveticus* R0052*, B. longum* R0175	1 sachet/day, 10 × 10^9^ CFU/sachet	28	Yes	Double-blind, randomized controlled trial	Male (29%), adults (36 ± 8), normal-weight	8 weeks	No	None	-	Depression	Yes	Kazemi *et al*. 2019
*Lb. johnsonii* LA1	2 sachets/day, 2 × 10^9^ CFU/sachet	48	Yes	Double-blind, randomized, placebo-controlled trial	Male (54%), adults (32 ± 10), BMI not reported	24 weeks	No	None	-	Recurrence of Chron's disease after surgery	No	Marteau *et al*. 2006
*Lb. johnsonii* LA1	1 sachet/day, 1 × 10^10^ CFU/sachet	27	Yes	Double-blind, randomized, placebo-controlled trial	Male (56%), adults (39 ± 15), BMI not reported	12 weeks	No	None	-	Recurrence of Chron's disease after surgery	No	Van Gossum *et al*. 2007
*Lb. paracasei* subsp. *paracasei* W8	1 capsule/day, 1 × 10^9^ OR 1 × 10^10^ CFU/capsule	21	Yes	Three arms, randomized, controlled, crossover study	Male (52%), adults (27 ± 7), normal-weight	3 days	No	None	-	Energy intake	No	Toksvig Bjerg *et al*.^2014^
*Lb. plantarum* ATTC 202 195	1 capsule/day, 1 × 10^9^ CFU/capsule	2185	Yes	Double-blind, randomized, placebo-controlled trial	Infants (0–2 months); sex not reported	8 weeks	No	None	-	Neonatal sepsis	Yes	Panigrahi *et al*. 2017
*Lb. plantarum* CCFM8610	1 serving/day, 1 × 10^9^ CFU/serving	29	Yes	Placebo-controlled trial	Male (40 or 38%), adults (49 ± 15 or 53 ± 14), normal-weight	8 weeks	No	16S rRNA and groEL gene sequencing	Increase in microbial diversity, Bacteroidetes and *B. pseudocatenulatum*, decrease in Firmicutes/Bacteroidetes ratio	Atopic dermatitis	Yes	Fang *et al*. 2019
*Lb. plantarum* ECGC 13 110 402	2 capsules/day, 2 × 10^9^ CFU/sachet	23	Yes	Prospective, randomized, placebo-controlled, parallel-group study	Male (22%), adults (52 ± 11), over-weight	12 weeks	Yes, 4 weeks	16S rRNA gene sequencing	None	Blood lipids	Yes	Costabile *et al*. 2017
*Lb. plantarum* Lp115	80 ml/day, 1.25 10^7^ UFC/ml	12	Yes	Not reported	Male (0%), adults (62 ± 4), over-weight	12 weeks	No	None	-	Metabolic syndrome in postmenopausal	Yes	Barreto *et al*. 2014
*Lb. plantarum* MF1298	1 capsule/day, 1 × 10^10^ CFU/capsule	16	Yes	Double-blind, randomized, placebo-controlled trial	Male (31%), adults (50 ± 11), normal-weight	16 weeks	No	None	-	IBS symptoms	No	Farup *et al*. 2012
*Lb. plantarum* PS128 (DSM 28 632)	1 capsule/day, 3 × 10^10^ CFU/capsule	36	Yes	Double-blind, randomized, placebo-controlled trial	Male (100%), children (10 ± 2), normal-weight	4 weeks	No	None	-	Autism spectrum disorder	Yes	Liu *et al*. 2019
*Lb. reuteri* ATTC PTA 6475	2 servings/day,5 × 10^9^ CFU/serving	45	Yes	Double-blind, randomized, placebo-controlled trial	Male (0%), adults (76 ± 1), normal-weight	48 weeks	No	None	-	Bone loss in old women	Yes	Nilsson *et al*. 2018
*Lb. reuteri* DSM 17 938	5 drops/day, 0.2 × 10^8^ CFU/drop	14	Yes	Double-blind, randomized, placebo-controlled trial	Male (43%), infants (8 ± 3 months)	4 weeks	No	None	-	Crying time in infants with colic	No	Nation *et al*. 2017
*Lb. reuteri* DSM 17 938	5 drops/day, 0.2 × 10^8^ CFU/drop	25	Yes	Double-blind, randomized, placebo-controlled trial	Male (60%), infants (1.2 ± 0.8 months)	3 weeks	No	FISH for specific targets	None	Crying time in infants with colic	Yes	Savino *et al*. 2010
*Lb. reuteri* DSM 17 938	5 drops/day, 0.2 × 10^8^ CFU/drop	32	Yes	Double-blind, randomized, placebo-controlled trial	Male (41%), infants (48 ± 26 months)	4 weeks	No	Real-time qPCR for specific targets	None	Crying time in infants with colic	Yes	Savino *et al*. 2018
*Lb. reuteri* DSM 17 938	5 drops/day, 0.2 × 10^8^ CFU/drop	85	Yes	Double-blind, randomized, placebo-controlled trial	Male (44%), infants (1.9 ± 0.7 months)	4 weeks	No	None	-	Crying time in infants with colic	No	Sung *et al*. 2014
*Lb. reuteri* DSM 17 938	1 serving/day, 1 × 10^8^ CFU/serving	15	Yes	Double-blind, randomized, placebo-controlled trial	Male (60%), infants (1 ± 0.4 months)	3 weeks	No	16S rRNA gene sequencing	None	Crying time in infants with colic	No	Roos *et al*. 2013
*Lb. reuteri* DSM 17 938	1 tablet/day, 1 × 10^8^ CFU/tablet	15	Yes	Double-blind, randomized, placebo-controlled, cross-over trial	Male (70%), adults, normal-weight	24 weeks	No	16S rRNA gene sequencing	Increase in microbial diversity and in Firmicutes	None	-	del Campo *et al*. 2014
*Lb. reuteri* DSM17938	1 serving/day, 10^8^ OR 10^10^ CFU/serving	15 OR 14	Yes	Double-blind, randomized, placebo-controlled, parallel-group study	Male (80%), adults (65 ± 6), BMI not reported	12 weeks	No	16S rRNA gene sequencing	None	Glucose metabolism in type-2 diabetes patients	No	Mobini *et al*. 2017
*Lb. reuteri* NCIMB 30 242	2 servings/day, from 3 × 10^9^ to 10 × 10^9^ CFU/serving	10	No	Randomized, dose-escalation design trial	Adults, normal-weight to obese; sex and age not reported	4 weeks	No	16S rRNA gene sequencing	None	Reduction of cholesterol	Yes	Martoni *et al*. 2015
*Lb. rhamnosus* 14E4	1 serving/day, 1 × 10^10^ CFU/serving	14	Yes	Double-blind, randomized, placebo-controlled, cross-over trial	Male (50%), adults (31 ± 4), normal-weight	2 weeks	Yes, 1 week	16S rRNA gene sequencing	Increase in *Prevotella* and decrease in *Bacteroides*	None	-	Bautista-Gallego *et al*. 2019
*Lb. rhamnosus* GG	1 serving/day, 4.5 × 10^9^ CFU/serving	15	Yes	Double-blind, randomized, placebo-controlled trial	Male (47%), infants (1.3 ± 0.4 months)	4 weeks	No	None	-	Crying time in infants with colic	No	Pärtty *et al*. 2015
*Lb. rhamnosus* GG	1 serving/day, 6 × 10^9^ CFU/serving	10	Yes	Double-blind, randomized, placebo-controlled trial	Male (59%), children (7 ± 2), BMI not reported	4 weeks	No	DGGE and FISH for specific targets	Increase in *Bacteroides*	Inflammatory status in cystic fibrosis children	Yes	Bruzzese *et al*. 2014
*Lb. rhamnosus* GG	1 bottle/day, 1.55 × 10^11^ CFU/bottle	10	Yes	Double-blind, randomized, placebo-controlled trial	Male (30%), adults (47 ± 6), normal-weight	3 weeks	No	Phylogenetic microarray	Increase in *Lb. rhamnosus*	None	-	Lahti *et al*. 2013
*Lb. rhamnosus* GG	1 serving/day, 1 × 10^9^ CFU/serving	8	Yes	Double-blind, randomized, placebo-controlled trial	Infants (6 months); sex not reported	24 weeks	No	Phylogenetic microarray	None	None	-	Cox *et al*. 2010
*Lb. rhamnosus* GG	4.5 × 10^7^–8.5 × 10^7^/g; day amount not reported	12	Yes	Double-blind, randomized, placebo-controlled trial	Male (75%), infants (1–12 months)	24 weeks	No	16S rRNA gene sequencing	Increase in *Blautia, Roseburia, Coprococcus*	Oral tolerance in cow's milk allergic children	Yes	Berni Canani *et al*. 2016
*Lb. rhamnosus* GG*, B. animalis* sub. *lactis* BB-12	1 capsule/day, 1 × 10^9^ CFU/capsule	207	Yes	Double-blind, randomized, placebo-controlled trial	Male (0%), adults (32 ± 4.8), from normal-weight to obese	8 weeks	No	None	-	Gestational diabetes	No	Callaway *et al*. 2019
*Lb. rhamnosus* GG, *Lb. rhamnosus* LC705, *B. breve* Bb99, *Propionibacterium freudenreichii* subsp. *shermanii* JS	1 capsule/day, 5 × 10^9^ CFU/capsule	461	Yes	Double-blind, randomized, placebo-controlled trial	Male (50%), children (1.2 ± 0.3)	24 weeks	No	None	-	Allergic diseases	No	Kukkonen *et al*. 2007
*Lb. rhamnosus* GG, *Lb. rhamnosus* Lc705, *Propionibacterium freudenreichii* subsp*. shermanii* JS*, B. animalis subsp. lactis* Bb12	120 ml/day, 1 × 10^7^ CFU/ml	43	Yes	Double-blind, randomized, placebo-controlled trial	Male (9%), adults (50 ± 13), normal-weight	20 weeks	No	Phylogenetic microarray	None	IBS symptoms	Yes	Kajander *et al*. 2007
*Lb. rhamnosus* IMC 501, *Lb. paracasei* IMC 502	1 serving/day, 1 × 10^9^ CFU/serving	25	Yes	Double-blind, randomized, placebo-controlled trial	Adults; sex, age and BMI not reported	12 weeks	Yes, 2 weeks	Microbial counts	Increase in lactobacilli and bifidobacteria	Bowel habits	Yes	Verdenelli *et al*. 2011
*Lb. salivarius* CECT5713	2 capsules/day, 1 × 10^8^ CFU/capsule	20	Yes	Double-blind, randomized, placebo-controlled trial	Male (50%), adults (33 ± 8), BMI not reported	4 weeks	No	Microbial counts	Increase in lactobacilli	Immune response	Yes	Sierra *et al*. 2010
*Lb. salivarius*, strain not reported	1 serving/day, 2 × 10^10^ CFU/serving	27	Yes	Double-blind, randomized, placebo-controlled trial	Adults (25 ± 5), normal-weight, sex not reported	16 weeks	No	None	-	Upper respiratory tract infections occurrence in athletes	No	Gleeson *et al*. 2012
*Lb. casei;* strain not reported	1 bottle/day, 6.5 × 10^9^ CFU/bottle	6	No	Not reported	Male (33%), children (13 ± 3), normal-weight	6 weeks	No	Intergenic spacer profiling (IS-pro)	None	None	-	El Manouni el Hassani *et al*. 2019
*Leuconostoc holzapfelii*; strain not reported	Not provided	21	No	Not reported	Male (43%), adults (28 ± 2.3), normal-weight	4 weeks	No	16S rRNA gene sequencing	None	None	-	Yang *et al*. 2019
*S. thermophilus, Lb. acidophilus, B. longum;* strains not reported	3 capsules/day, 3 × 10^10^ cfu/capsule	16	Yes	Double-blind, randomized, placebo-controlled trial	Male (69%), adults (54 ± 11), normal-weight	12 weeks	No	DGGE	None	Chronic kidney disease	No	Borges *et al*. 2018
Supherb Bio-25–*B. bifidum, Lb. rhamnosus, Lc. lactis, Lb. casei, B. breve, S. thermophilus, B. longum* sub. *longum, Lb. paracasei, Lb. plantarum* and *B. longum* sub. *infantis;* strains not reported	2 pills/day, 2.5 × 10^10^ CFU/pill	14	Yes	Placebo-controlled trial	Male (57%), adults (42 ± 13), normal-weight	4 weeks	No	Shotgun metagenome, real-time qPCR	Probiotic strains engraftment is dependent on individualized gut microbiome features	None	-	Zmora *et al*. 2018
Symbiter*—Lactobacillus* + *Lactococcus, Bifidobacterium, Propionibacterium, Acetobacter;* species and strains not reported	10 g/day, 6 × 10^10^ CFU/g	31	Yes	Double-blind, randomized, placebo-controlled trial	Adults (52 ± 2), obese, sex not reported	8 weeks	No	None	-	Insuline resistance in type-2 diabetes patients	Yes	Kobyliak *et al*. 2018a
Symbiter—see above	10 g/day, 6 × 10^10^ CFU/g	30	Yes	Double-blind, randomized, placebo-controlled trial	Adults (53 ± 10), obese, sex not reported	8 weeks	No	None	-	Non-Alcholic Fatty Liver Disease	Yes	Kobyliak *et al*. 2018b
Symprove—*Lb. rhamnosus* NCIMB 30 174*, Lb. plantarum* NCIMB 30 173*, Lb. acidophilus* NCIMB 30 175, *Enterococcus faecium* NCIMB 30 176	1 serving/day, 1 × 10^10^ CFU/serving	100	Yes	Double-blind, randomized, placebo-controlled trial	Male (32%), adults (39 ± 11), BMI not reported	12 weeks	No	None	-	IBS symptoms	Yes	Sisson *et al*. 2014
Vivomixx ®—*Lb. plantarum* DSM 24 730, *S. thermophilus* DSM 24 731, *B. breve* DSM 24 732, *Lb. paracasei* DSM 24 733, *Lb. delbrueckii* subsp. *bulgaricus* DSM 24 734, *Lb. acidophilus* DSM 24 735, *B. longum* DSM 24 736, *B. infantis* DSM 24 737	2 sachets/day, 4.5 × 10^11^ CFU/sachet	9	Yes	Double-blind, randomized, placebo-controlled trial	Male (100%), adults (45 ± 10), BMI not reported	24 weeks	No	None	-	Neuroinflammation in HIV patients	Yes	Ceccarelli *et al*. 2017
VSL#3®—*S. thermophilus, B. breve, B. longum, B. infantis, Lb. acidophilus, Lb. plantarum, Lb. paracasei, Lb. delbrueckii*; strains not reported	2 sachets/day, 1.1 × 10^11^ CFU/sachets	14	No	Open-label, single-arm study	Male (0%), adults, age and BMI not reported	4 weeks	Yes, 4 weeks	16S rRNA gene sequencing	None	Immune response	Yes	Singh *et al*. 2018
VSL#3®—see above	1 capsule/day, 1.1 × 10^11^ CFU/capsule	15	Yes	Randomized, placebo-controlled trial	Adults (50 ± 10), overweight or obese; sex not reported	6 weeks	No	Microbial counts	None	Cholesterol in obese subjects	Yes	Rajkumar *et al*. 2014
VSL#3®—see above	1 capsule/day, 1.1 × 10^11^ CFU/capsule	15	Yes	Double-blind, randomized, placebo-controlled trial	Adults, sex, age and BMI not reported	8 weeks	No	Phylogenetic microarray	None	Diarrhea-predominant IBS synthomps	Yes	Michail and Kenche 2011
VSL#3®—see above	2 sachets/day, 9 × 10^11^ CFU/sachets	10	No	Open-label	Male (20%), adults (46 ± 10), BMI not reported	4 weeks	No	16S rRNA gene sequencing	Decrease in *Bacteroides*	IBS symptoms	Yes	Ng *et al*. 2013
VSL#3®—see above	1.1 × 10^11^ CFU/sachets; day amount not reported	20	No	Not reported	Male (65%), adults (44 ± 27), BMI not reported	4 weeks	No	None	-	Liver cirrosis	Yes	Marlicz *et al*. 2016
VSL#3®—see above	2 sachets/day, 1.1 × 10^11^ CFU/sachets	22	Yes	Double-blind, randomized, placebo-controlled trial	Male (45%), children (10 ± 2), obese	16 weeks	No	None	-	Non-Alcholic Fatty Liver Disease in obese children	Yes	Alisi *et al*. 2014
VSL#3®—see above	1.1 × 10^11^ CFU/sachets; day amount not reported	9	Yes	Double-blind, randomized, placebo-controlled trial	Male (33%), adolescents (14 ± 2), obese	16 weeks	No	16S rRNA gene sequencing	None	Obesity	No	Jones *et al*. 2018
VSL#3®—see above	1 sachet/day, 9 × 10^11^ CFU/sachet	66	Yes	Double-blind, randomized, placebo-controlled trial	Male (85%), adults (48 ± 5), BMI not reported	24 weeks	No	None	-	Recurrence of hepatic encephalopathy in patients with cirrhosis	Yes	Dhiman *et al*. 2014
Yakult—*Lb. casei* Shirota	1 bottle/day, 6.5 × 10^9^ CFU/bottle	10	Yes	Double-blind, randomized, placebo-controlled trial	Adults; age, sex and BMI not reported	20 weeks	No	None	-	Immune response in allergic rhinitis	Yes	Ivory *et al*. 2008
Yakult—*Lb. casei* Shirota	1 bottle/day, 1 × 10^11^ CFU/bottle	23	Yes	Double-blind, randomized, placebo-controlled trial	Male (52%), adults (23 ± 0.4), normal-weight	8 weeks	No	16S rRNA gene sequencing	None	Stress-induced abdominal dysfunction	Yes	Kato-Kataoka *et al*. 2016
Zircombi—*B. longum* BB536*, Lb. rhamnosus* HN001	1 sachet/day, 4 × 10^9^ CFU/sachet	23	Yes	Double-blind, cross-over, randomized, placebo-controlled trial	Male (17%), adults (48 ± 3), normal-weight	4 weeks	Yes, 2 weeks between treatments	16S rRNA gene sequencing	Increase in *Bifidobacterium*, decrease in *Enterobacter, Klebsiella, Serratia*	Lactose intolerance	Yes	Vitellio *et al*. 2019
*Lb. johnsonii* LA1	80 ml/day, 1 × 10^7^ CFU/ml	74	Yes	Double-blind, cross-over, randomized, placebo-controlled trial	Male (57%), children (12 ± 2), BMI not reported	3 weeks	No	None	-	*Helicobacter pylori* infection eradication	Yes	Gotteland *et al*. 2008

*As reported in the original study.

In some cases, independent clinical trials tested the efficacy of the same probiotic formulation targeting an identical health outcome (Table 1). For example, Chahwan *et al*. (2019), Steenbergen *et al*. (2015) and colleagues administered a multistrain probiotic preparation (Ecologic® Barrier) containing 4 strains of *Lactobacillus* spp., 2 strains of *Lc. lactis*, and 3 strains of *Bifidobacterium* spp. to adults with depression, and both studies reported an improvement in neurocognitive functions and relief from the symptoms of depression. In contrast, the same formulation was not effective at improving blood glycaemia in type 2 diabetes patients (Horvath *et al*. 2019). This highlights that a probiotic formulation should be designed to target a well-defined health outcome or population. In two other trials, the consumption of *Lb. johnsonii* LA1 for 12 or 24 weeks was proposed to reduce the recurrence of Crohn's disease after surgery (van Gossum *et al*. 2007; Marteau *et al*. 2006). The investigators considered two different doses (4 × 10^9^ or 1 × 10^10^ CFU/day), but the treatment was shown to be ineffective in both trials.

In other cases, the results obtained in separate trials were discordant. The same strain of *Lb. reuteri* (DSM 17 938) was independently examined for its activity against infant colic in five trials, all with similar duration (2–3 weeks) and number of cells ingested per day (10^8^ CFU/day). However, only two of the trials reported a reduction in crying time (Savino et al. 2010, 2018; see Table 1).

Notably, none of the studies presented in this review commented on the rationale for choosing a specific probiotic dosage. The ISAPP provides suggested amounts ranging from 1 × 10^8^ to 1.8 × 10^12^ CFU once or twice a day, depending on the strain and the type of target population (Guarner *et al*. 2012). However, this list covers gastrointestinal disorders only and does not take into account the intrinsic variability in the gut microbiota. In general, different dosages should be assessed to understand the effect of the dose-response relationship of probiotic consumption on targeted health outcomes and support the causality of the observed associations. This might at least partly explain the presence of contrasting results observed in clinical trials found in the literature.

Contrasting results might also be related to a subject-specific response associated with individual characteristics of the gut microbiome. In fact, several studies reported that different individuals may respond differently to the same drug, dietary treatment or even probiotic treatment and that this difference may be at least partially related to the individual structure of the gut microbiome (Zmora *et al*. 2016; De Filippis et al., 2018). According to some recent studies, the baseline microbiome may also influence the possibility of the probiotic strain colonizing the gut and its long-term engraftment and persistence once oral administration is stopped (Maldonado-Gomez *et al*. 2016; Zmora *et al*. 2018). Nevertheless, most of the studies found in the literature did not explore the effect of probiotic administration on the gut microbiota composition (Table 1). Moreover, even when this analysis was included, the method used did not allow tracking of the fate of the specific strain except in a few cases (Suez *et al*. 2018; Zmora *et al*. 2018). Indeed, probiotic treatment was frequently not able to modify the overall structure of the gut microbiota, and only a few studies reported an increase in the abundance of the microbial genus/genera administered. Finally, interpretation of the effect of probiotic consumption on the composition of the gut microbiota may be particularly complicated due to the lack of consensus around a universally accepted definition of a healthy gut microbiota (Bäckhed *et al*. 2012; Cani 2018; McBurney *et al*. 2019).

As reported above, some LAB strains may exhibit health-promoting activity even if inactivated. Some clinical trials demonstrated that the consumption of fermented matrices in which probiotic bacteria were heat-inactivated may still have a positive effect on health (Table 1). For example, consumption of milk fermented by *Lb. paracasei* CBA L74 and subsequently heat-killed reduced the occurrence of infectious diseases in children and boosted the production of beneficial short-chain fatty acids (Berni Canani *et al*. 2017; Corsello *et al*. 2017). In another trial conducted in an adult population, the consumption of inactivated *Lb. gasseri* CP2305 in tablets reduced anxiety and stress (Nishida *et al*. 2019).

## FERMENTED FOODS AND HUMAN HEALTH



Growing evidence has been provided in the literature regarding the enhanced functional and nutritional properties of FFs. Notably, a large proportion of such foods contain living microorganisms, including LAB, which are genetically similar to the strains used as probiotics. Extensive clinical trials have been conducted to prove the human health-promoting activities of probiotics, as reviewed in the previous section. Epidemiological and clinical studies on FFs have also been similarly conducted, although there are considerably fewer such studies, and a large fraction of them have been well reviewed in (Marco *et al*. 2017; Fernandez and Marette 2018; Kok and Hutkins 2018).

Consumption of fermented dairy products was inversely correlated with overall mortality (Soedamah-Muthu *et al*. 2013) and impaired glucose metabolism (Eussen *et al*. 2016) and linked to overall improvements in health-linked biomarkers (González *et al*. 2019). Yogurt consumption was inversely associated with type 2 diabetes (Chen *et al*. 2014; Díaz-López *et al*. 2016), risks of metabolic syndrome (Babio *et al*. 2015), risk of colorectal cancer (Pala *et al*. 2011), and long-term weight gain (Mozaffarian *et al*. 2011). Multiple randomized, controlled trials showed a higher effectiveness of probiotic yogurts than conventional yogurts in the improving various health outcomes, such as fasting blood glucose levels (Ejtahed *et al*. 2012) and insulin resistance (Asemi *et al*. 2013; Nabavi *et al*. 2015). Different species and strain types present in fermented milk were related to reduced muscle soreness (Iwasa *et al*. 2013), improved intrinsic brain activity (Tillisch *et al*. 2013), reduced incidence of fever (Nagata *et al*. 2016), improved bowel movements (Nagata *et al*. 2016), reduced risk of cardiovascular disease (Sonestedt *et al*. 2011), beneficial effects on metabolism (Fernandez and Marette 2018), and positive changes in health and mood (Baars 2019). High consumption of cultured milk lowered the risk of developing bladder cancer (Larsson *et al*. 2008). Combined consumption of cheese, yogurt, and fermented milk was inversely associated with diabetes (Sluijs *et al*. 2012). Consumption of fermented kimchi decreased insulin resistance, increased insulin sensitivity (An *et al*. 2013) and reduced the prevalence of asthma and atopic dermatitis (Park and Bae 2016; Kim, Ju and Park 2017). In a randomized controlled study, kimchi also improved fasting blood glucose levels and other health outcomes (Kim *et al*. 2011; Choi *et al*. 2013). Kefir-fermented milk was associated with a large six-month increase in hip bone mineral density among osteoporotic patients (Tu *et al*. 2015), and Chungkookjang improved multiple parameters associated with obesity (Byun *et al*. 2016). The risk of high blood pressure was reduced by the consumption of fermented products such as miso and natto (Nozue *et al*. 2017). Total cholesterol levels were improved by consumption of fermented soy (Cavallini *et al*. 2016) and Kochujang (Lim *et al*. 2015). Signs of irritable bowel syndrome were attenuated by consumption of low-FODMAP rye bread (Laatikainen *et al*. 2016) and lacto-fermented sauerkraut (Nielsen *et al*. 2018). Clinical trials on FFs have also been conducted in animal models. Examples in mice are represented by the alleviation of atopic dermatitis through cream cheese (Kim, Kim and Kim 2019) and the recovery from antibiotic-induced gut dysbiosis with gut barrier function enhancement through a mixture of *Lactobacillus* species isolated from traditional FFs (Shi *et al*. 2018). Focusing specifically on the impact of FFs on gastrointestinal health, there is still limited evidence of the effectiveness of these foods (Mota de Carvalho *et al.*^2018^; Dimidi *et al*. 2019; Wu *et al*. 2019). Kefir is the FF most commonly investigated in this scenario, with evidence of a beneficial role for lactose malabsorption and *Helicobacter pylori* eradication (Dimidi *et al*. 2019). In addition, a recent observational study carried out in the frame of the American Gut project, highlighted that fermented plant consumers showed higher faecal levels of beneficial conjugated linoleic acid (CLA) compared with non-consumers, besides increased abundance of several LAB species and other health-associated taxa (e.g. *Faecalibacterium prausnitzii, Prevotella* spp., *Eubacterium* spp.) in their gut microbiome. Interestingly, CLA dietary intake was not different between the two groups, suggesting an effect of the gut microbiome in the biosynthesis (Taylor *et al*. 2020).

## CONCLUSIONS

FFs are widespread worldwide and can be considered as primary reservoirs of live bacteria that can potentially reach the gut microbiome and eventually impact host health. Although many speculations about the food-gut axis exist, there is only limited evidence supporting a direct effect of FF consumption on the gut microbiome and on the possible transfer of LAB from FFs to the host gut microbiome. Well-designed clinical trials and broad population studies are necessary to understand whether ingested LAB are effectively able to engraft in the human gut and become permanent members of the gut microbiome. As discussed, the ability of certain strains to reach and colonize the human gut will strongly depend on their genomic capacity to counteract the barriers that they will face (i.e. low pH and bile salts), as well as the specific composition of the host gut microbiome. In future years, probiotic research will surely benefit from recent advances in genomics and metagenomics and the development of new bioinformatic algorithms and analysis tools. To develop a robust knowledge of the LAB-food-gut axis, comparative genomic studies will be needed to compare the diversity and functions of LAB from food and gut environments. In addition, improved availability of LAB genomes from gut isolates will also be important for better understanding the genomic features (if any) that can be pivotal for the adaptation of LAB to the gut environment. Meanwhile, FFs will continue to serve as inexhaustible sources of potential probiotic LAB strains and as natural dietary sources of live LAB cells with a promising role in human gut health.

## Supplementary Material

fuaa015_Supplemental_Files
